# A ribosome-interacting jumbophage protein associates with the phage nucleus to facilitate efficient propagation

**DOI:** 10.1371/journal.ppat.1012936

**Published:** 2025-02-24

**Authors:** Wichanan Wannasrichan, Sucheewin Krobthong, Chase J. Morgan, Emily G. Armbruster, Milan Gerovac, Yodying Yingchutrakul, Patompon Wongtrakoongate, Jörg Vogel, Chanat Aonbangkhen, Poochit Nonejuie, Joe Pogliano, Vorrapon Chaikeeratisak

**Affiliations:** 1 Department of Biochemistry, Faculty of Science, Chulalongkorn University, Bangkok, Thailand; 2 Center of Excellence in Natural Products Chemistry (CENP), Department of Chemistry, Faculty of Science, Chulalongkorn University, Bangkok, Thailand; 3 School of Biological Sciences, University of California San Diego, La Jolla, California, United States; 4 Institute for Molecular Infection Biology (IMIB), Faculty of Medicine, University of Würzburg, Würzburg, Germany; 5 Helmholtz Institute for RNA-based Infection Research (HIRI), Helmholtz Centre for Infection Research (HZI), Würzburg, Germany; 6 Helmholtz Centre for Infection Research (HZI), Braunschweig, Germany; 7 National Center for Genetic Engineering and Biotechnology, NSTDA, Pathum Thani, Thailand; 8 Department of Biochemistry, Faculty of Science, Mahidol University, Bangkok, Thailand; 9 Center of Excellence on Petrochemical and Materials Technology, Chulalongkorn University, Bangkok, Thailand; 10 Center for Advanced Therapeutics, Institute of Molecular Biosciences, Mahidol University, Salaya, Nakhon Pathom, Thailand; Montana State University, UNITED STATES OF AMERICA

## Abstract

Bacteriophages must hijack the gene expression machinery of their bacterial host to efficiently replicate. Recently, we have shown that the early-expressed protein gp014 of *Pseudomonas* nucleus-forming phage phiKZ forms a stable complex with the host ribosomes and modulates the overall protein expression profile during phage infection. Here, we discover a nucleus-forming phage, designated Churi, that is closely related to phiKZ. Churi encodes gp335, a homolog of gp014-phiKZ, which is expressed during the early stages of infection, and its overexpression in bacterial cells interferes with bacterial growth, suggesting its role in phage-host interplay. We predict experimentally that gp335 also interacts with host ribosomal proteins, similar to its homolog gp014-phiKZ, thereby strengthening its involvement in protein translation during phage infection. We further show that GFP-tagged gp335 specifically localizes by clustering around the phage nucleus and remains associated with it throughout the infection cycle. The CRISPR-Cas13-mediated deletion of gp335 reveals that the mutant phage fails to replicate efficiently, resulting in an extended latent period. Altogether, our study demonstrates that gp335 is an early-expressed protein of the Chimallivirus Churi that localizes in proximity to the phage nucleus, likely serving a role in localized translation to ensure efficient phage propagation.

## Author summary

Bacteriophages are prokaryotic viruses that infect bacteria. Due to their relatively small genome size, they typically rely on their host’s machinery for reproduction. Recently, we discovered nucleus-forming jumbophages that harbor large genomes, displaying intricate organization via nucleus-based replication. Despite their unique subcellular organization through the formation of a nucleus-like structure, similar to other phages, their protein translation machinery remains dependent on the bacterial host. In this study, we identified a novel nucleus-forming jumbophage, Churi, and demonstrated that it produces an early-expressed protein, gp335, which likely interacts with the host ribosomes. Additionally, gp335 localizes specifically near the nuclear shell, where ribosomes are presumably situated, throughout the infection cycle, likely playing a role in the translation of phage proteins on the nuclear surface. In the absence of gp335, the phage exhibits reduced reproductive efficiency, leading to slower infection and delayed cell lysis. This phage-host interplay is conserved in other nucleus-forming phages and might have evolutionary implications.

## Introduction

Bacteriophages are increasingly being considered as a promising alternative to cope with the antimicrobial resistance crisis [1] due to their abundance in nature and efficacy against both naïve pathogenic and antibiotic-resistant bacteria [2,3]. During propagation within bacterial cells, most phages rely on various host factors, including proteins involved in DNA replication, RNA transcription, protein translation, and energy metabolism. As a result, they often encode several proteins that hijack these mechanisms and optimize the intracellular environment to facilitate phage progeny production. These proteins often target essential host proteins involved in fundamental metabolism [4]. Numerous studies have demonstrated that most of these proteins are expressed early during phage infection, and several can interfere with bacterial growth after induction [5–11]. For example, *Pseudomonas* phage LUZ24 produces a small polypeptide called Igy, that specifically binds to the host DNA gyrase. The Igy protein exhibits antimicrobial activity against *Pseudomonas aeruginosa* in both PAO1 and PA14 strains when expressed within bacterial cells because it interrupts host DNA gyrase activity leading to the collapse of DNA replication and cell death [8,9]. Furthermore, some phages have been found to interfere with host RNA transcription [12]. Phage LUZ19 encodes a protein, gp25.1 that interacts with the β´-subunit of *P. aeruginosa* RNA polymerase. This protein inhibits host transcription and reduces bacterial growth [6,11]. However, the proteins mentioned earlier are mainly found in small-genome phages. The proteins of jumbophages, which are used to hijack bacterial cells and tune the cellular metabolism into a favourable environment for phage propagation, remain largely unexplored.

Unlike other phages, jumbophages typically possess numerous genes that facilitate their replication within bacterial cells [13]. They tend to rely on their own proteins. For example, nucleus-forming jumbophages, also known as Chimalliviruses, produce a nucleus-like structure [14,15] that functions as a protective barrier, shielding phage DNA from bacterial immune systems [16], and also creates a specialized compartment for phage DNA replication [15]. In addition, Chimalliviruses encode their own DNA polymerase, DNA helicase, and RNA polymerase, which are essential for phage DNA replication and RNA transcription inside the nucleus-like structures [14]. Despite this self-sufficiency, these phages still require host translation machinery for their protein synthesis, and the proteins involved in this process are not well-characterized [4,17]. Recently, our research group identified an early-expressed protein of the nucleus-forming phage phiKZ, gp014, that interacts with the ribosomes of *P. aeruginosa*. This protein directly binds to the large ribosomal subunit of *P. aeruginosa* and plays a regulatory role in translation by binding in close proximity to 5S rRNA [17]. Even though the mutant lacking gp014 does not impair phage propagation in the laboratory strain *P. aeruginosa* PAO1, it does alter the overall protein expression pattern, which may impact phage fitness during its replication cycle in this particular strain [17].

In this study, we identify Churi as a previously uncharacterized nucleus-forming jumbophage that is closely related to phiKZ and demonstrate that it also encodes a homolog of gp014-phiKZ, gp335-Churi, as an early gene product. gp335-Churi strongly interferes with the growth of *P. aeruginosa*, when overexpressed from a plasmid, suggesting its role in the phage-host interplay during host takeover. Co-immunoprecipitation assays demonstrate that gp335-Churi also interacts with bacterial ribosomes, similar to what was previously observed in gp014-phiKZ. We further reveal that, during Churi infection, gp335-Churi accumulates around the phage nucleus and remains associated with the nucleus throughout the infection. A loss-of-function mutant of gp335-Churi, which gp335 fails to localize near the phage nucleus, exhibits a delay in bacterial cell lysis, and complementation of the wild-type gp335-Churi can recover the latent period to wild-type timing. This study of the nucleus-forming jumbophage Churi reveals an early gene product that associates with the phage nucleus, likely interacting with the host ribosomes to translate phage proteins and maintain efficient phage replication.

## Results

### Churi is classified in *Chimalliviridae* and relies on the nucleus-based replication

**Fig 1:**
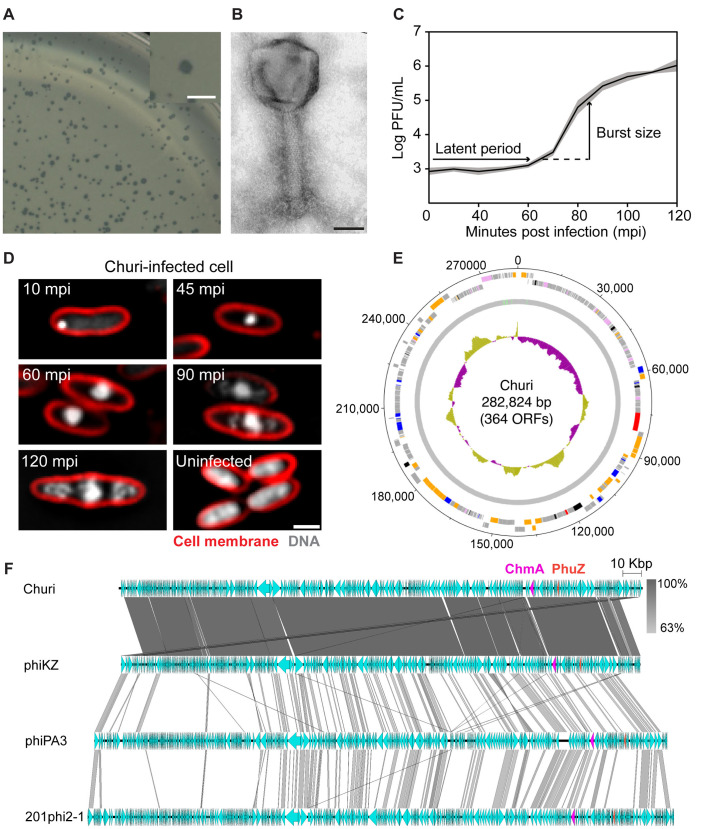
Characteristics of phage Churi reveal that it is a Chimallivirus. (A) Double layer agar shows plaques morphology of Churi, scale bar represents 2 mm; (B) A transmission electrogram reveals Churi particle morphology, which is categorized as a myovirus, and scale bar represents 50 nm. (C) One-step growth curve of Churi. (D) Single cell infection against *P. aeruginosa* of Churi under fluorescence microscope. FM4-64 (red) stains the bacterial cell membrane while DAPI (gray) binds the DNA. A scale bar represents 1 µm. (E) Circular genomic map of phage Churi. ORFs indicated by colors as follows: blue, DNA replication, transcription and translation and DNA repair; pink, nucleotide metabolism and modification; orange, virion structural and assembly; red, host-phage interaction and host lysis protein; black, others; and gray, hypothetical proteins. An inner plot presents the GC content across the genome (yellow above average and purple below average). The numbers surrounding the map represent nucleotides. The map was generated using Artemis: DNAPlotter version 18.1.0. (F) Comparative genomic analysis shows the gene organization of phage Churi in comparison with phiKZ, phiPA3, and 201phi2-1. Of note, the genomes of phiKZ, phiPA3, and 201phi2-1 are presented in the reverse direction to improve data clarity. Orange-labelled arrows represent the location and orientation of PhuZ, while magenta arrows represent shell (ChmA).

To isolate a jumbophage, a soil sample was collected from the area of Chulalongkorn University, and the phages within the sample were enriched against *P. aeruginosa* PAO1. The supernatant from the enrichment culture was plated on a *P. aeruginosa* cell lawn, and small plaques, characteristic of jumbophage morphology, were selected and purified. This process led to the isolation of a clonal phage, which we have designated as Churi. Churi exhibits a plaque morphology that is clear throughout the plaque area with rough borders and has a plaque size ranging from 1 to 1.5 mm in diameter on 0.35% LB agar (Fig 1A). Negative staining and electron microscopy revealed that Churi is a myophage. The total length of a Churi particle, from the top of the capsid to the bottom of the base plate, is approximately 300 nm, with the icosahedral capsid measuring about 120 nm in both length and width. The contractile tail measures approximately 140 nm in length and 25 nm in width (Fig 1B). The latent period of phage Churi is approximately 65 minutes post-infection (mpi), at which point the number of phage progenies steeply rises. The average burst size of phage Churi is around 59 particles per cell (Fig 1C).

We then performed a single-cell level infection assay to monitor the progression of phage infection within the bacterial host cell. The result revealed a bright punctum of DAPI-stained DNA located at the cell pole around 10 minutes post-infection (mpi). As the infection progresses, the DNA punctum increases in size and migrates toward the cell center by 45 mpi (Figs 1D **and** S1). This spatiotemporally organized replication machinery observed in Churi appears analogous to the nucleus-based replication observed in chimalliviruses, which are capable of assembling a phage nucleus during their lytic cycle [14,15,18]. Like chimalliviruses, during late infection, DAPI-stained horseshoe-shaped structures, also known as phage bouquets [19], which are composed of mature virions, assemble and localize at the periphery of the central DNA at 90 mpi. These structures become larger and brighter at 120 mpi, indicating the accumulation of mature capsids. The proportion of infected cells containing bouquets increases throughout the infection period, from 70% at 90 mpi to approximately 90% at 120 mpi. (Figs 1D **and** S1). The frequency of bouquet formation in Churi is notably similar to that observed in other chimalliviruses, comparable to phiPA3 (~80%). However, the frequency in Churi is significantly higher compared to phiKZ (~23%) and 201phi2-1 (~2%), suggesting a differing degree of subcellular organization for phage maturation among these phages [19].

The complete genome of Churi was obtained through whole genome sequencing. The genome topology of Churi is double-stranded DNA, which is circularly permuted and terminally redundant. The genome size is 282,824 base pairs with a low GC content of 36.9%, and it encodes 364 open reading frames (ORFs) (Fig 1E). Churi is classified as a jumbophage due to its genome size exceeding 200 kb [13]. The genome encodes 7 tRNAs responsible for 6 amino acids: threonine, leucine, proline, isoleucine, aspartic acid, and asparagine. Among all ORFs, 282 ORFs encode hypothetical proteins of unknown function, while 82 ORFs can be functionally annotated. The genes with known functions are involved in various biological processes as followings: virion structural components and assembly (42 genes); DNA replication, transcription and translation, and DNA repair (14 genes); nucleotide metabolism and modification (14 genes); host-phage interaction and host lysis (2 genes); and miscellaneous functions (10 genes). Importantly, the nuclear shell protein ChmA and the phage tubulin PhuZ are conserved in the Churi genome, consistent with other well-studied chimalliviruses (Fig 1F**; magenta and orange arrows)**. This conservation indicates a shared genotype among bacteriophages within the family *Chimalliviridae*, which encompasses all known nucleus-forming phages [14], thereby confirming Churi as a member of the chimallivirus group. VIRIDIC analysis [20] comparing Churi and other well-studied chimalliviruses further demonstrates that Churi is most closely related to phiKZ and OMKO1 (another phiKZ-like phage), with 94.2% intergenomic sequence similarity (S1 Table**).** However, these phages are not classified as the same viral species according to the demarcation criteria of ICTV, which specify that different phage species should possess less than 95% genome sequence identity [20,21]. In contrast, the sequence similarity of Churi to phiPA3 and 201phi2-1 is relatively low, at 18.3% and 12.3%, respectively. Despite the high similarity in genomic organization between Churi and phiKZ, there are some ORFs around 10 kilobases that are differently oriented among their genomes. Furthermore, fewer genes were found to be conserved between Churi, phiPA3, and 201phi2-1 compared to phiKZ (Fig 1F).

### The early-expressed gp335 of Churi interferes with bacterial growth and interacts with host translation machinery

It has been long known that most bacteriophages do not possess all enzymes required for their gene expression and, therefore, must co-opt with the host gene expression machinery during their replication cycle [4,22,23]. Although chimalliviruses often have their own non-virion RNA polymerase to facilitate transcription [13,24,25], like other phages, they remain entirely dependent on the host’s protein translation machinery. Given our hypothesis that phage-host interactions begin immediately upon phage infection of the bacterial host cell, we conducted mass spectrometry on cultures during the early stages of infection, within the first 15 minutes, to identify phage proteins that may be involved in the phage-host interplay. We harvested infected cells from 2 infection methods: plate cultures and liquid cultures, at 15 mpi. While 31 non-virion phage proteins were identified in plate infection cultures (S2 Table) and 129 non-virion phage proteins in liquid infection cultures (S3 Table), only 19 proteins were consistently detected in both experiments, confirming the presence of these phage-expressed proteins regardless of the infection model (S2 and S3 Tables**; Grey-shaded rows)**. gp270, which is a homolog of gp068-phiKZ, a component of the non-virion RNA polymerase complex (nvRNAP), was also detected in our experiment (S2 and S3 Tables). Since nvRNAP is recognized as one of the early phage genes [26,27], these data support the validity of our proteomic results in identifying early proteins expressed by Churi. Among the consensus proteins, 15 hypothetical proteins were selected for further experiments (Table 1).

**Table 1:** Selected non-structural proteins of Churi found to express at 15 minutes post-infection from both proteomic experiments and their homologs in other nucleus-forming phages (phiKZ and phiPA3).

Proteins	Function	Homologs	
		phiKZ	phiPA3
gp005	Hypothetical protein	gp293.1	–
gp059	Hypothetical protein	gp248	gp303
gp094	Hypothetical protein	gp219	–
gp110	Hypothetical protein	gp206	–
gp123	Hypothetical protein	gp194	gp227
gp130	Hypothetical protein	gp187	–
gp135	Hypothetical protein	gp183	–
gp150	Hypothetical protein	gp168	gp197
gp177	Hypothetical protein	gp145	gp164
gp199	Hypothetical protein	gp124	gp140
gp256	Hypothetical protein	gp077	gp074
gp279	Hypothetical protein	gp059	gp055
gp325	Hypothetical protein	–	gp115
gp335	Hypothetical protein	gp014	gp122
gp354	Hypothetical protein	gp304	gp376

**Fig 2:**
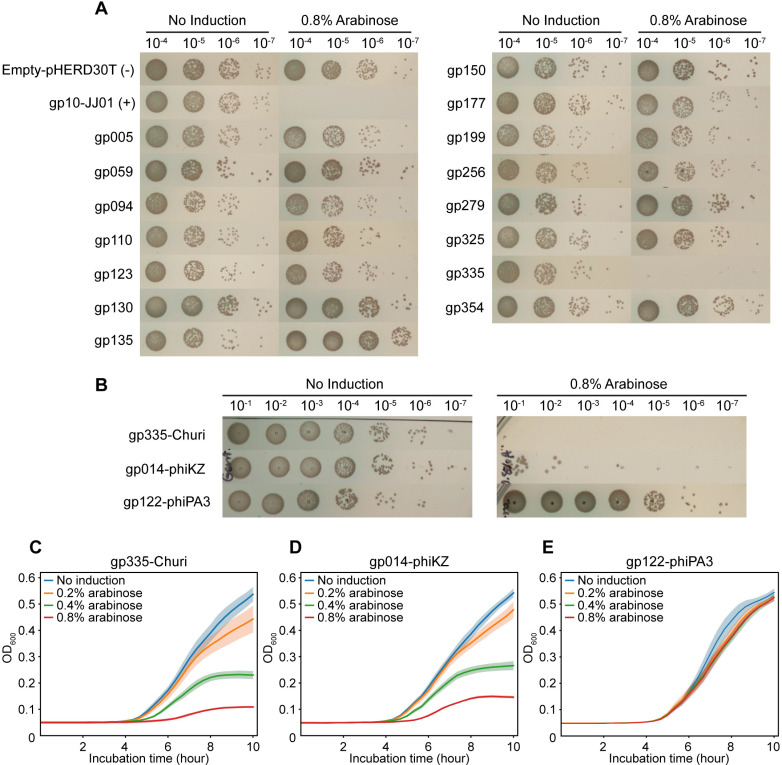
Growth inhibition assay of Churi- protein expressing *P. aeruginosa* reveals that gp335 substantially interferes with the bacterial growth. (A) Churi proteins that have inhibitory activity against *P. aeruginosa* PAO1 were screened from 15 proteins found to express in the early infection of both proteomic experiments. Empty-pHERD30T represents negative control while gp10-phage JJ01 is used as the positive control of growth inhibitory protein. Ten-fold dilution of protein-expressing bacterial culture is spotted on LB agar supplemented with gentamicin. The spots are shown as the dilution from 10^−4^ to 10^−7^. (B) Inhibitory activity of gp335-Churi homologs in phiKZ and phiPA3. The spots are shown as the dilution from 10^−1^ to 10^−7^. All recombinant strains of *P. aeruginosa* are induced with or without 0.8% arabinose. (C)-(E) Optical density (OD_600_) of bacteria expressing gp335-Churi compared to gp014-phiKZ, and gp122-phiPA3 when the cells were induced with different concentration of arabinose (0.2, 0.4, and 0.8%). The OD_600_ values were measured every 20 minutes for 10 hours of incubation. Shaded error bar represents standard deviation (±SD) of n=6. (C) gp335-Churi, (D) gp014-phiKZ, and (E) gp122-phiPA3.

With the aim to investigate phage proteins that intercept with host cellular processes, we reasoned that if phage proteins interfere with the host gene expression apparatus, overexpression of these proteins in the cell could disrupt essential cellular machinery, necessary for the bacterial cell growth, subsequently leading to bactericidal or bacteriostatic effects. We then performed a growth inhibition assay to assess the impact on *P. aeruginosa* cell growth upon overexpression of early-expressed phage proteins, aiming to identify those that engage with the host’s gene expression machinery. We tested the 15 candidate proteins for growth inhibition against *P. aeruginosa* PAO1 by inducing phage gene expression using pHERD30T plasmids with arabinose. An empty pHERD30T was used as a negative control, while gp10 of phage JJ01 [28], whose homolog was previously identified as a strong bacterial growth inhibitor [6], served as a positive control. The growth of bacteria containing the empty pHERD30T plasmid (negative control) did not decrease at the highest concentration of inducer (arabinose), while gp10-JJ01 (positive control) effectively suppressed growth in an arabinose concentration-dependent manner, confirming that the expression system functioned as expected and that growth suppression was specific to the expressed protein (Figs 2 **and** S2). Among the fifteen candidates, only gp335 significantly reduced the growth of *P. aeruginosa* when its protein level was overproduced with arabinose. The reduction in colony-forming units (CFUs) was at least 6 log compared to the non-induction culture (Fig 2A **and** 2B). We further explored whether this inhibitory activity of gp335-Churi is also conserved in its homologs in other closely related *Pseudomonas* chimalliviruses (gp014-phiKZ: 99.73% similarity, and gp122-phiPA3: 28.41% similarity). Interestingly, only gp014-phiKZ exhibited the same inhibitory activity against *P. aeruginosa* PAO1 as gp335-Churi, whereas gp122-phiPA3 did not (Fig 2B–E). The bacterial CFU count also sequentially decreased with increasing concentrations of arabinose induction of gp335-Churi (S2 Fig), suggesting that the growth inhibition activity of gp335-Churi against *P. aeruginosa* is dose-dependent. The real-time effects of gp335-Churi on *P. aeruginosa* growth were also observed by measuring optical density (OD) in liquid culture at various induction levels. Over a 10-hour growth period, 0.2% arabinose induction slightly reduced optical density, 0.4% induction significantly reduced optical density, and 0.8% induction almost completely inhibited culture growth (Fig 2C**).** Likewise, this growth pattern was also observed in cells expressing gp014-phiKZ (Fig 2D). In contrast, no difference in bacterial growth was observed between uninduced and induced conditions for gp122-phiPA3, similar to the negative control (Figs 2E **and** S2). These data suggest that the conserved functions of the homologs in Churi (gp335) and phiKZ (gp014) are likely to involve interference with cellular processes essential for cell growth.

To further elucidate potential interaction partners of gp335 in *P. aeruginosa* during the phage-host interplay, we used gp335-sfGFP as a bait, expressing it in *P. aeruginosa*, and specifically pulled it down using an anti-GFP antibody, followed by subsequent analysis via LC-MS/MS for protein identification. sfGFP alone served as an internal control for gp335-sfGFP, and the proteins pulled down with sfGFP were subtracted from the list to finalize the putative molecular interactors of the Churi gp335 protein (S4 Table). Among the 20 highly scored proteins (Table 2), three putative interacting partners are involved in bacterial RNA transcription, including the top-scoring interacting partner, the transcriptional regulator RtcR, and six are ribosomal proteins involved in protein translation. These include 50S ribosomal proteins L22, L28, and L4, and 30S ribosomal proteins S9, S4, and S5. This finding is consistent with our recent report showing that gp014-phiKZ, the homolog of gp335-Churi, interacts with the *P. aeruginosa* ribosomes [17]. Our previous study of gp014-phiKZ strengthens the current data on gp335-Churi, further suggesting that these proteins share a conserved function in interacting with host ribosomes to facilitate phage-host interactions during early infection. Given our findings that gp335-Churi is an early-expressed phage product that should also interact with host ribosomes, based on its near-identical nature to gp014-phiKZ, we sought to determine how this interaction benefits the phage replication cycle. This formed the basis of our experiments to further investigate the spatiotemporal organization of gp335 during phage Churi infection and how gp335 is necessary for phage Churi propagation.

**Table 2:** List of first twenty *P. aeruginosa* PAO1 proteins according to Sequest HT (abundant) score from co-immunoprecipitation (Co-IP) experiment with gp335-sfGFP.

Accession	Description	Coverage (%)	No. of Peptides	No. of PSMs	No. of amino acid	Score Sequest HT
Q9HVK3	**Transcriptional regulator RtcR**	4	1	49	531	126.97
Q9HVT2	Alpha-2-macroglobulin homolog	1	1	43	1516	113.16
Q9HWK6	Lysyl endopeptidase	16	5	23	462	57.25
Q9I0R3	Probable chemotaxis transducer	4	1	23	535	56.5
Q9I2Y0	**Probable transcriptional regulator**	3	1	20	901	54.3
Q9HW00	Probable peptidoglycan glycosyltransferase FtsW	7	1	15	399	44.61
Q9HWE0	**50S ribosomal protein L22**	12	1	14	110	41.42
Q9HVY3	**30S ribosomal protein S9**	32	3	16	130	40.61
Q9I6N6	**Probable transcriptional regulator**	16	1	15	158	36.43
Q9HXZ3	tRNA(Ile)-lysidine synthase	6	1	13	442	32.52
Q9HTY4	Acetylornithine deacetylase	7	1	12	384	32.49
Q9I194	Acyl-homoserine lactone acylase PvdQ	2	1	14	762	29.23
Q9HTN8	**50S ribosomal protein L28**	13	1	11	78	25.03
Q9I4U5	Antitoxin Xre/MbcA/ParS-like toxin-binding domain-containing protein	22	1	9	95	23.45
Q9HWU9	Probable dehydrogenase	13	1	7	229	20.15
Q9HWD6	**50S ribosomal protein L4**	24	2	6	200	20.04
O52759	**30S ribosomal protein** **S4**	9	1	6	206	17.05
Q9HWF2	**30S ribosomal protein** **S5**	17	2	7	166	16.67
P05384	DNA-binding protein HU-beta	32	2	6	90	16.29
Q9HVY8	Stringent starvation protein B	19	1	5	135	14.2

### gp335 accumulates around and remains associated with the phage nucleus throughout the Churi infection cycle

**Fig 3:**
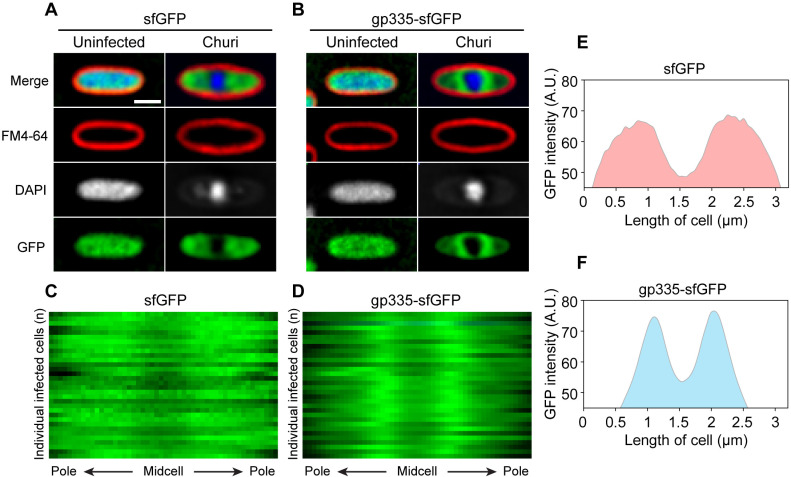
GFP-tagged gp335 accumulates close to the phage nucleus during Churi infection. Localization of GFP-tagged proteins, induced with 0.2% arabinose, when *P. aeruginosa* K2733 was infected with Churi at 90 mpi compared to uninfected control, (A) sfGFP and (B) gp335-sfGFP. Scale bar represents 1 µm. Kymograph shows the distribution of GFP intensity of individual infected cells (n = 32) at 90 mpi, (C) sfGFP control and (D) gp335-sfGFP. Distribution plots corresponding to the kymograph show the different pattern of GFP intensity between (E) sfGFP control and (F) gp335-sfGFP. A.U. means arbitrary units.

The reproduction of Chimalliviruses relies upon a protein-based nuclear structure that partitions proteins according to their functions and serves as a site for DNA packaging during phage maturation [14–16,18,24,29,30]. The phage nucleus also uncouples and segregates RNA transcription and protein translation within bacterial cells. mRNA is transcribed from the phage genome inside the phage nucleus and must be exported to the cell cytoplasm, where ribosomes are located to facilitate translation into proteins. Since gp335 was experimentally predicted to bind to the host ribosomes, we further investigated the localization of gp335 relative to the phage nucleus and its spatiotemporal organization in bacterial cells during phage infection. The result revealed that, in the absence of phage infection, gp335-sfGFP is uniformly distributed throughout the cells, similar to the control sfGFP alone (Fig 3A and 3B**; uninfected)**, suggesting that gp335 is soluble in the cytoplasm and does not specifically localize within bacterial cells. During infection at 90 mpi, when the phage nucleus is fully mature and positioned at the midcell, gp335-sfGFP is excluded from the phage nucleus, similar to sfGFP alone (Fig 3A and 3B**; Churi)**. This indicates that gp335 is not selectively transported into the phage nucleus. Interestingly, gp335-sfGFP accumulates around the phage nucleus, assembling a cloud-like pattern in the Z-section (Fig 3B**; Churi)**. Kymographs depicting 1-pixel horizontal slices from the central axis of 32 infected cells further illustrate that, unlike the sfGFP control, which is diffused from pole to pole and excluded from the phage nucleus (Fig 3C), the GFP intensity of gp335-sfGFP is the highest adjacent to the phage nucleus at the midcell (Fig 3D). Correspondingly, the GFP intensity plot confirms the distribution of sfGFP along the cell length (Fig 3E), while the intensity plot of gp335-sfGFP shows narrow peaks close to the phage nucleus (Fig 3F). These data suggest that gp335 specifically localizes close to the nuclear shell, where ribosomes are likely translating mRNAs that are exported across the shell.

**Fig 4:**
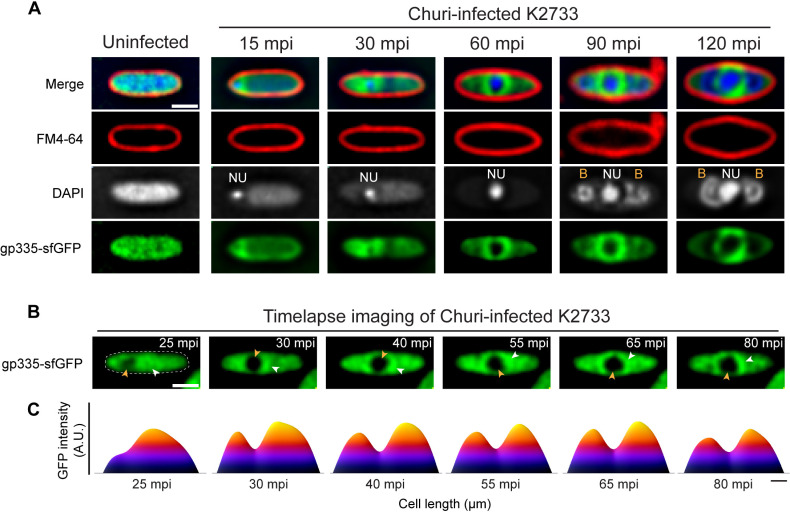
gp335 accumulation appears around the phage nucleus at early infection and moves along with the nucleus as Churi infection progresses. (A) Time-series images show the localization of GFP-tagged gp335 (green) during Churi infection against *P. aeruginosa* K2733 from early to late time point (minutes post-infection, mpi) compared to an uninfected cell. NU and B are abbreviations for the phage nucleus and bouquets, respectively. (B) Time-lapse images show the single cell infection of Churi against *P. aeruginosa* K2733 expressing GFP-tagged gp335. Yellow arrowhead indicates the location of the phage nucleus while the white arrowhead indicates the area of GFP with dense intensity. Scale bars in A and B represent 1 µm. (C) GFP intensity heatmap of gp335 corresponding to the time-lapse imaging using raw images reveals the movement and accumulation of gp335 along with the Churi nucleus. Scale bar in **C** represents 0.5 µm. A.U. means arbitrary units.

To determine the spatiotemporal dynamics of gp335 localization over time, we performed time-series imaging on Churi-infected cells expressing gp335-sfGFP and visualized the protein localization as the infection progressed. We found that, at 15 mpi, the DAPI-stained early phage nucleus appears near the cell pole with gp335-sfGFP accumulating adjacent to it. As the nucleus moves towards the cell center at 30 mpi, the gradient of gp335-sfGFP moves along with it and remains associated with the phage nucleus. The cloud of gp335-sfGFP becomes denser and forms a ring-like morphology in the Z-section, encircling the phage nucleus at 60 mpi. This structure increases in diameter as the phage nucleus grows in size from 60 to 120 mpi (Figs 4A **and** S3). Later in the infection, phage bouquets assemble, as visualized by DAPI staining, and these structures exclude gp335-sfGFP, similar to previous observations in other nucleus-forming phages (Fig 4A) [19]. Time-lapse imaging and fluorescence intensity plots of a single-infected cell over the course of the infection cycle confirm the spatiotemporal dynamics of gp335, which remains associated with the phage nucleus throughout the infection (Fig 4B and 4C). Combined with our finding that gp335 is an early-expressed protein and probably interacts with the host ribosomes, these data further suggest that gp335 rapidly accumulates around the early phage nucleus and remains associated with it throughout the replication cycle, potentially facilitating localized protein translation near the nuclear shell.

### gp335 is not essential but required to localize near the phage nucleus for efficient phage replication

**Fig 5:**
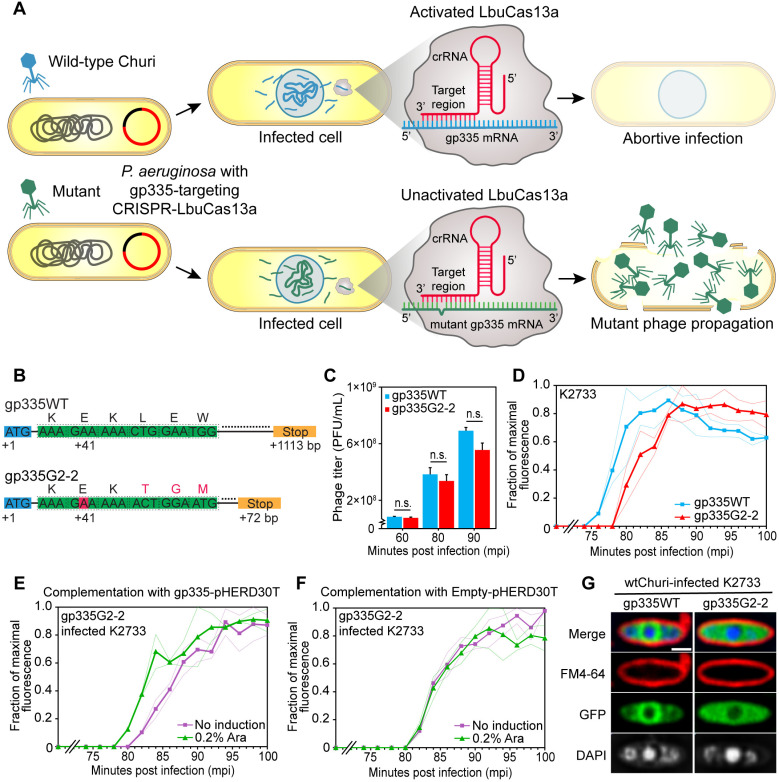
gp335-mutant Churi slightly delays in cell lysis and complementation of gp335 improves time to lysis of the mutant phage. (A) The schematic mechanism of mutant phage isolation with LbuCas13a. An anti-sense guide RNA complementarily pairs with the mRNA transcript of gp335. This activates the Cas13a that leads to abortive infection of Churi bearing the wild-type gp335. The mutant phages, that already exist as the sub-population escape this selection and become major or dominant populations. (B) Variant calls on gp335 from whole genome sequence of wild-type (gp335WT) and mutant gp335G2-2 Churi are shown as a gene diagram indicating a nucleotide insertion at early sequence of gp335 (red box). This leads to frameshift mutation (red alphabets) and stop codon at +72 bp. Numbers represent nucleotide position in the gene. Orange box is a stop codon. (C) Titer count (PFU/mL) in different minutes post-infection between wild-type (gp335WT) and gp335G2-2 mutant Churi as determined by spot test on *P. aeruginosa* K2733 lawns. n = 3 per condition. n.s. means non significance at *p ≤ 0.05* (paired-samples *t*-test, SPSS). (D)-(F) Single-step time-to-lysis represents real-time bacterial cell lysis caused by the phage infection. Phage progenies, that are released from infected cells, are detected by SYTOX green. (D) Lysis pattern of wild-type Churi compared to mutant gp335G2-2. Complementation experiment shows lysis time of (E) mutant gp335G2-2 Churi against *P. aeruginosa* expressing gp335 (induced with or without 0.2% arabinose) while (F) empty-pHERD30T was used as a control to ensure that arabinose does not affect the lysis of Churi. Solid lines depict the mean across n ≥ 4 replicates. Faint lines show mean +/- standard deviation. (G) Fluorescence images between *P. aeruginosa* K2733 cells that expressed either wild-type gp335-sfGFP or gp335G2-2-sfGFP when they were infected with wild-type Churi at 90 mpi. Scale bar represents 1 µm.

To determine if gp335 is essential for Churi replication, we utilized *Leptotrichia buccalis* CRISPR-Cas13a (Cas13) with a guide RNA targeting gp335 to select for naturally occurring mutant phages with mutations in gp335 (Fig 5A). Since Cas13 provides a strong selective pressure against the targeted sequence, only phages with mutations in that sequence can propagate; otherwise, infection would be abortive. If gp335 was essential, we would expect to find only mutants with silent or missense mutations that might retain gp335 function. However, if gp335 was non-essential, nonsense mutations could be present. Our genomic sequencing of derived mutants revealed that gp335 is non-essential, as indicated by the presence of nonsense mutations early in the gene. A mutant, designated gp335G2-2, was selected for further study as it contained an insertion of adenine (A) at position +41 base pair of gp335, which caused a frameshift mutation and an early stop codon. This mutation shortened the gene to only 72 bp compared to the wild-type gene (1,113 bp), with only the first 15  translated amino acids remaining similar to the wild-type; subsequent residues were translated out-of-frame until amino acid position 23 (Fig 5B). In contrast, when we previously used this method to select for mutants of the essential gene gp069-phiKZ, only missense or silent mutations were identified [30]. Thus, the nonsense mutation observed in gp335G2-2, which causes a frameshift and premature stop codon resulting in significant alterations to the gp335-Churi product, indicates that gp335 is not essential for Churi viability.

Consistent with our findings from mutant screening, a previous study on gp014-phiKZ [17], the homolog of gp335-Churi, also demonstrated that this protein is not essential for phage replication in *P. aeruginosa* strain PAO1, as the phiKZ mutant lacking gp014 could still produce progeny at levels comparable to the wild-type phage. By evaluating progeny production through phage titer measurement of the gp335 mutant compared to the wild-type, our data in this study further confirm the non-essential function of gp335 in Churi within the same laboratory strain. Our results showed that the phage titer of the gp335G2-2 mutant was lower than that of the wild-type (gp335WT), but the difference was not statistically significant (Fig 5C).

Additionally, we previously demonstrated that, even though gp014-phiKZ is not essential, the phiKZ mutant lacking gp014 exhibits an altered overall protein expression pattern in bacterial host [17]. This suggests a regulatory role of gp014 in the synthesis of phage proteins. We hypothesized that this altered profile of phage protein expression might affect the robustness of the phage replication cycle, potentially resulting in an extended latent period. To further examine whether the loss-of-function of gp335 impairs the phage fitness and leads to a prolonged latent period, we performed the single-step time-to-lysis assay to compare the latent periods between the wild-type and gp335G2-2 mutant Churi. This technique is highly accurate for measuring bacterial cell lysis, as it uses a cell-impermeable fluorescent DNA staining dye that stains released nucleic acids upon cell lysis, rapidly increasing in brightness for detection [31]. We found that, as determined by the point at which 50% of maximal fluorescence is reached, the wild-type Churi lyses the cell on average at 78 mpi, while the gp335G2-2 mutant lyses at 82 mpi (Fig 5D). This indicates a delay in bacterial cell lysis when infected with the mutant. Complementation of the infection by Churi gp335G2-2 with the wild-type gp335 protein expressed from pHERD30T after induction was able to rescue the replication cycle of the mutant, reducing the latent period by up to 4 minutes compared to the uninduced control. This further suggests that, while gp335 is not essential for phage replication, it plays a role in maintaining phage fitness to regulate the appropriate latent period of the replication cycle (Fig 5E). Neither uninduced nor induced empty-pHERD30T with arabinose had any effect on the lysis timing of the gp335G2-2 mutant (Fig 5F).

To further investigate whether the inactive mutant gp335 expressed by Churi gp335G2-2 retains its localization, we created a C-terminal fusion of this mutant gp335 with sfGFP and expressed it to visualize the localization of the mutant during infection. The result revealed that, unlike the wild-type protein, the mutant protein fails to localize close to the nuclear shell, and no ring-like pattern was observed around the phage nucleus in the mutant (Fig 5G). Altogether, these data suggest that gp335 is required to accumulate close to the phage nucleus, potentially to facilitate rapid localized protein translation and ensure proper proteome expression patterns, thereby maintaining efficient reproduction timing.

## Discussion

Jumbophages have garnered significant interest recently, not only due to their intricate replication strategies [13,15,18,24,25,29,30,32,33], but also for their potential applications in therapy and biocontrol [34–38]. This interest is partly attributed to the presence of a substantial number of uncharacterized genes that facilitate their invasion and regulation within bacterial hosts [13,14,16]. In this study, we first isolated a jumbophage and successfully obtained a new *P. aeruginosa* jumbophage, Churi. Genomic analysis revealed that Churi is closely related to phiKZ, representing a variant of the over 30 different phiKZ-like phages that have been reported in the Genbank database to date. Churi’s genome encodes homologs of ChmA (a major component of the phage nuclear shell), PicA (involved in protein import into the phage nucleus), and PhuZ (orchestrating nuclear positioning and capsid trafficking), which are conserved among nucleus-forming phages [14,30]. Additionally, the morphology and protein localization pattern of Churi-infected cells are consistent with those of other nucleus-forming phages, as evidenced by microscopy data (Figs 1D **and** S1). Altogether, these genomic analysis and microscopic data suggest that Churi is a previously unidentified member of the phiKZ-like phages within the *Chimalliviridae* family [14].

Despite the overall genomic similarity between Churi and phiKZ, not all genes are conserved. Approximately 4% of Churi’s genes exhibit low conservation, with less than 80% identity to phiKZ. These include gp056-phiKZ and gp283-Churi (HNH nuclease; 35.1% identity), gp094-phiKZ and gp238-Churi (internal head protein; 40% identity), gp303-phiKZ and gp355-Churi (hypothetical protein; 70.1% identity), and gp299-phiKZ, previously found to interacting with 50S ribosomal subunit [17], and gp358-Churi (hypothetical protein; 69.5% identity). Moreover, some genes are unique to each phage. For example, gp001, gp208, gp209, gp325, and gp340 (Methyltransferase type 11) are unique in Churi and absent in phiKZ, while gp072 (HNH endonuclease) and gp296 of phiKZ are not found in Churi. These distinct genotypes suggest species-specific differences that may influence the unique phage-host interactions between these bacteriophages. In addition to genomic differences, the replication machinery within the host also displays some distinctions. Even though Churi is closely related to phiKZ, the frequency of bouquet formation during late infection in Churi is more similar to phiPA3, which is more distantly related. Bouquets are observed in Churi in around 70% of infected cells at 90 mpi (S1 Fig), compared to only 20% in phiKZ and 80% in phiPA3 [19]. Further investigation into the genomic differences between Churi and phiKZ, including their encoded products, may shed light on the factors essential for jumbophage bouquet assembly.

Recently, a number of studies have reported that bacteriophages express a variety of proteins during early infection, which target key components of bacterial cells [5–11]. For instance, early-expressed proteins from phiKZ (gp058, gp124, and gp287) have been identified to bind to bacterial DnaX and FtsZ, although neither interacts with protein translation machinery of *P. aeruginosa* [6]. Our proteomic analysis of Churi revealed several early-expressed gene candidates, among which gp335 was identified as a factor that intercepts with the host gene expression machinery, and its overexpression interferes with *P. aeruginosa* growth. Our finding here agrees well with our previous study on gp014-phiKZ, the homolog of gp335-Churi [17]. However, in this study, we further demonstrate that in addition to gp014-phiKZ’s role in modulating the overall phage protein expression pattern [17], overproduction of gp014-phiKZ also affect bacterial growth, similar to gp335-Churi (Fig 2B and 2D), suggesting a conserved role for these 2 homologs. Given that gp014-phiKZ levels plateau at approximately 2,000 copies per cell during phiKZ infection (which is fewer than the total number of cellular ribosomes) [17,39], this inhibitory effect on bacterial growth may result from the overexpression of gp014-phiKZ, which potentially saturates host ribosomes and consequently disturbs the bacterial proteome. It is not surprising that the activity of both gp335-Churi and gp014-phiKZ is similar, as the amino acid sequence of gp335-Churi shares 99.73% identity with that of gp014-phiKZ (S4 Fig **and** S5 Table). In contrast, gp122-phiPA3, which shares only 28.41% amino acid identity with gp335-Churi, does not exhibit this growth interference property (Fig 2B and 2E **and** S5 Table). We previously elucidated that gp014-phiKZ requires certain conserved amino acids for complete interaction with ribosomes, including R10, K15, W18, R23, K152, R262, and K263 [17]. Multiple alignment of gp335 and its homologs, gp014-phiKZ and gp122-phiPA3, revealed that even though most residues are conserved across all 3 phages, K15, W18, and K152 are not conserved in gp122-phiPA3 (S4 Fig). It is important to note that homologs in nucleus-forming phages can still perform comparable functions, despite sharing low sequence identity. For example, PhuZ proteins in nucleus-forming phages (201phi2-1, phiKZ, and phiPA3) exhibit dynamic instability during phage infection despite sharing only 30–40% amino acid sequence similarities [18,29]. Based on this evidence, we remain convinced that gp122-phiPA3 may possess a function comparable to those of Churi and phiKZ. Further investigation into the role of these less conserved residues (K15, W18, and K152) in gp122-phiPA3 could provide insights into the regulation of protein synthesis during phiPA3 infection. Additionally, gp110-Churi (Table 1), a homolog of gp206-phiKZ, which has been reported to co-sediment with the 50S ribosomal subunit [17], does not inhibit cell growth, unlike gp335-Churi (Fig 2A). This observation suggests a more critical role for gp335 in the regulation of phage gene expression compared to other ribosome-associating phage proteins.

Throughout Churi infection, gp335 is spatiotemporally localized adjacent to the phage nucleus. At 15 mpi, a gradient of gp335 forms and accumulates at the immature phage nucleus near the cell pole. This finding complements previous research showing that gp014-phiKZ is rapidly recruited to an early phage infection (EPI) vesicle, which assembles during the early stages of the nucleus-forming phage infection cycle (10 mpi), likely playing a key role in the translation of early-expressed phage proteins [40,41]. Our Co-IP results for gp335-Churi in this study reveal a possible interaction with host ribosomes. Based on the near-identical nature between gp335-Churi and gp014-phiKZ, this interaction is supported by our previous single-particle cryogenic electron microscopy (cryo-EM) of gp014-phiKZ, which validated the binding of this protein to the bacterial large ribosomal subunit at high resolution [17]. In this study, we further demonstrated that throughout the infection, gp335 consistently accumulates around the phage nucleus and moves along with it but does not enter the nucleus. Given the proteins it interacts with, this suggests that gp335 is not involved in DNA replication or transcription [15] but rather in the translation of mRNA exported from the phage nucleus, which must be in close proximity to the nuclear shell. The active mechanisms responsible for maintaining the location of gp335 close to the phage nuclear shell remain unclear. Additionally, we identified host transcriptional regulators as potential molecular interactors of gp335-Churi, suggesting that gp335 may also co-opt *P. aeruginosa* transcription during Churi infection*.* Further investigation into the role of gp335 in transcriptional interference will be required.

Cas13 selection of gp335-null Churi mutants revealed that gp335 is non-essential for phage propagation. However, the loss-of-function of gp335, which results in the failure to maintain its correct subcellular localization, causes a delay in the lytic life cycle of Churi. This prolonged latent period can be rescued by exogenous expression of the wild-type gp335. This finding suggests that gp335 and its association with the phage nucleus are important for efficient Churi propagation within the host, possibly by maintaining phage fitness through proper protein expression. It is worth mentioning that the negative impact of this gp335 mutant is not as pronounced in the laboratory strain *P. aeruginosa* PAO1, similar to the gp014-phiKZ mutant [17]. However, the phiKZ mutant exhibits a more significant negative effect on phage propagation when infecting specific clinical isolates of *P. aeruginosa* [17]. To gain further insights into phage-host interplay with diverse bacterial hosts, additional investigation into the effect of gp335-Churi in other *P. aeruginosa* strains might be necessary. Overall, gp335-Churi is clearly beneficial for the efficient completion of the phage lytic cycle. This finding has evolutionary implications, as faster completion of the lytic cycle may provide phages with a selective advantage over slower competitors [42,43].

## Materials and methods

### Ethics statement

This work has been reviewed and approved by Chulalongkorn University-Institutional Biosafety Committee (CU-IBC) in accordance with the levels of risk in pathogens and animal toxins listed in the Risk Group of Pathogen and Animal Toxin (2017) published by Department of Medical Sciences (Ministry of Public Health), the Pathogen and Animal Toxin Act (2015) and Biosafety Guidelines for Modern Biotechnology BIOTEC (2016) with approval number: SC CU-IBC-028/2020 Ex1.

### Phage isolation, purification, and propagation

A soil sample collected from Chulalongkorn university, Bangkok, Thailand was enriched by adding LB medium, 0.5 mM CaCl_2_, and overnight culture of *P. aeruginosa* PAO1. The mixture was incubated at 37°C for 48 hours with shaking at 200 rpm. After the incubation, the phage-enriched culture was centrifuged at 8,500 rpm, 4°C for 10 minutes and filtered through 0.45 μm filter. Double-layer agar (DLA) plates were prepared by mixing 10 µL of enriched filtrates, 100 µL of *P. aeruginosa*, and 5 mL of 0.35% LB agar (Top agar). Then, the mixtures were transferred onto LB plates (Bottom agar). After incubation, single plaques were selected and kept in SM buffer. Plaques with the same morphology were selected at least 3–5 repeats to ensure that the isolated phages were purified. To propagate high titer bacteriophage, phage-containing SM buffer was diluted into the suitable dilution. At the concentration that web lysis in which plaques merged to another throughout the plate, appeared, the plates were soaked with 5 mL of SM buffer for 5 hours. After that, the phage lysate was collected and centrifuged at 8,500 rpm, 4°C for 10 minutes before filtration with a 0.45 µm-filter. The phage lysate was kept at 4°C, and phage titer was counted by spot test [18,28].

### One-step growth curve

*P. aeruginosa* PAO1 in exponential phase (OD600~0.4) was infected with phage Churi at MOI of 0.01 for 15 min at 37°C. Then, the cells were centrifuged at 8,500×g for 2 minutes. The pellet was washed with LB broth before centrifugation at 8,500×g for 2 minutes. After discarding supernatant, the pellet was resuspended with 10 mL of LB broth and incubated in a shaker at 250 rpm, 37°C for 2 hours. Every 10 minutes, including time point 0, the phage-bacterial mixture was collected and filtered with 0.45 µm filters. The filtrates were counted by spot titer assay. The PFU/mL will be calculated and plotted against minutes post-infection (mpi). The latent period was observed. Likewise, phage burst size was calculated from the plotted curve as particles/cell [34,44].

### Transmission electron microscopy (TEM)

Phage Churi lysate was precipitated with 10% (w/v) polyethylene glycol and 1 M NaCl overnight. The precipitated phage was centrifuged at 8,500 rpm for 10 minutes and resuspended with SM buffer. The phage was negatively stained with 2% uranyl acetate on a carbon-coated grid and the morphology of phages was visualized by transmission electron microscope (HITACHI model HT7700). Then, the phage classification was based on their structure according to ICTV classification observed under TEM.

### Phage genomic DNA extraction

Genomic DNA of phage Churi was extracted using phenol-chloroform–isoamyl alcohol method. Briefly, the phage lysate with at least 10^9^ PFU/mL was mixed with 10% (w/v) polyethylene glycol and 1M NaCl overnight to precipitate the phage particles. Phage precipitate was centrifuged at 8,500 rpm for 10 minutes [45] before resuspended with SM buffer. After that, host DNA and RNA were digested with 10 U of DNase I and 0.1 mg/mL of RNase A at 37°C. The DNase and RNase were inhibited by 20 mM EDTA. Then, the phage capsid was digested by 0.5 mg/mL of proteinase K and 0.5% sodium dodecyl sulfate (SDS). After the incubation at 55°C for 1 hour, Phenol-Chloroform–Isoamyl alcohol (25:24:1) (Sigma Aldrich, Switzerland) was added to the mixture at equal volume. The mixture was gently mixed before centrifuged at 15,000×g for 10 minutes and the top aqueous layer was collected. The steps of Phenol-Chloroform–Isoamyl alcohol and centrifugation were repeated before treated with 1/10 volume of 3 M sodium acetate and 2 volumes of cold absolute ethanol. The genomic DNA was allowed to precipitate at −20°C for 3 hours followed by centrifugation at 21,000×g for 20 minutes. The DNA pellet was washed with 70% ethanol, centrifuged at 15,000×g for 5 minutes, and the pellet was air-dried until the remaining ethanol evaporated. The phage DNA was dissolved with TE buffer. DNA quality and quantity were analysed using a NanoDrop 2000 spectrophotometer (Thermo Scientific) [28,46].

### Whole genome sequencing (WGS), genome analysis, and comparison

Whole genome sequencing was performed using the MiSeq (Illumina). FASTQ files with 2,110,430 reads of the Churi genome derived from the sequencing were assembled with Unicycler version 0.4.8 [47]. The single contig with coverage over 1,500x was then annotated with DNA Master version 5.23.6 (Glimmer 3.02 and GeneMark HMM). Functional prediction was manually performed using viral genomes available in the NCBI database, NCBI conserved domain, and Phyre2 were additionally used for the annotation. The phage genome map was constructed and visualized using Artemis: DNAPlotter version 18.1.0. Genetic relation and genome organization between phage Churi and other nucleus-forming jumbophages, including phiKZ (NC_004629.1), phiPA3 (NC_028999.1), and 201phi2-1 (NC_010821.1) were compared by EasyFig software version 2.1 [48]. Pairwise intergenomic similarities between related phage genomes were performed using the Virus Intergenomic Distance Calculator (VIRIDIC; http://rhea.icbm.uni-oldenburg.de/VIRIDIC/) [20]. The VIRIDIC algorithm relies on ICTV criteria to calculate intergenomic similarities among bacteriophages for classification.

### Proteomics study of phage Churi during infection

We performed proteomics study in different infection conditions. First is the infection on solid medium. Briefly, mid-log phase or OD_600_~0.6 (~6×10^8^ CFU/mL) of *P. aeruginosa* PAO1 was centrifuged at 4,500 rpm for 10 minutes. The pellet was resuspended with LB broth before spreading on LB plates and incubated for 5 hours. Then, high titer phage Churi was gently spread onto the plate with an MOI of 1. At 15 minutes post-infection, the bacteria-phage mixtures were flooded with LB broth and gently scraped with L-shape spreader before transferred into microcentrifuge tubes. The following steps were performed on ice or at 4°C. After centrifugation at 15,000xg for 1 minute, the supernatants were discarded and resuspended with 1X lysis buffer (2% SDS, 3 mM IAA, 1X RIPA buffer, and 1X Protease inhibitor cocktail). Samples were sonicated at 21% amplitude for 10 seconds with 1 second-pulse on/off and pelleted at 15,000×g for 1 min to remove the cell debris and intact cells [15]. The supernatants were immediately kept at −80°C. For the infection in liquid culture, log phase *P. aeruginosa* PAO1 was mixed with phage Churi at an MOI of 15 [49,50]. At 15 minutes post-infection, the mixtures were rapidly cooled on ice and collected at 5,000 rpm, 4°C for 5 minutes. The pellets were washed three times with ice-cold 1X PBS and resuspended in 500 µL of 1X lysis buffer. The samples on ice were sonicated at 21% amplitude for 3 minutes with 5 second-pulse on/off before centrifugation at 15,000×g for 1 minute to remove the cell debris and intact cells. The samples were kept at −80°C. Both solid and liquid infections were performed in duplicate. To analyze the phage Churi proteins expressed at 15 minutes post-infection, the protein concentration was measured before injected into LC-MS/MS. The LC-MS/MS analysis was performed at Department of Biochemistry, Faculty of Science, Mahidol university, Bangkok. Phage structural proteins were subtracted from all peptide signals that were above the cutoff value. Churi proteins found to be expressed in both solid and liquid-mediated infection were selected for further experiments.

### Growth inhibition assay of Churi candidate proteins

The selected non-structural genes of Churi that were expressed during the early infection were cloned into the *E. coli*-*Pseudomonas* shuttle vector pHERD30T [15] using Gibson assembly. The correctness of the recombinant plasmid was verified by Sanger sequencing. Then, the construct was electroporated into competent cells of *P. aeruginosa* PAO1. The recombinant cells were cultured in LB with high gentamicin selection (300 µg/mL) to keep the plasmid. To test the growth inhibition activity of Churi proteins, 200 µL of LB broth containing gentamicin with different concentrations of arabinose or without the arabinose was dispensed into 96-well plates. Overnight cultures of recombinant *P. aeruginosa* were diluted until OD_600_ ~0.2 and further 10-fold diluted. One microliter of each recombinant culture was added into the wells in triplicate. The 96-well plates were wrapped with parafilm to prevent evaporation and incubated at 37°C for 18 hours. After incubation, the culture from each well was 10-fold serially diluted and 5 µL of each diluted culture was spotted onto LB agar containing gentamicin. The plates were incubated at 37°C for 18 hours and colony-forming units were then calculated. Empty-pHERD30T and gp10 (JJ01)-pHERD30T were used as a negative control and positive control respectively.

Real-time activity against *P. aeruginosa* of phage proteins was also observed by measuring the optical density (OD_600_) of *P. aeruginosa* that expressed phage proteins. Bacterial cells were cultured overnight before the OD_600_ was adjusted to ~0.2, then 1 µL of each cultured were transferred into 96-well plates containing LB with gentamicin and different concentrations of arabinose from 0.2, 0.4, and 0.8%. The OD_600_ was measured every 20 minutes after gene induction for 10 hours at 37°C.

### Pulldown experiment for protein-protein interaction analysis

Overnight culture of gp335-sfGFP *P. aeruginosa* PAO1 was diluted to OD_600_~0.1. Then, the cells were further grown in 150 mL of LB broth supplemented with 25 µg/mL of gentamicin and 0.4% arabinose until OD_600_~0.3 followed by centrifugation at 4,000 rpm, 4°C, for 10 minutes. After that, the pellets were transferred into microcentrifuge tubes and then resuspended with 1 mL of lysis buffer (0.1% lysozyme, 25 mM Tris; pH 7.5, 150 mM NaCl, 4 mg/mL lysozyme, 20 µg/mL DNase I, 2X cOmplete protease inhibitor cocktail, and 0.4 mM PMSF) [51]. The reaction tubes were incubated at 37°C for an hour. The cells were further disrupted using probe tip sonication at a frequency of 20 kHz and 80% amplitude for 2 seconds on, and 8 seconds off, for the total of 20 seconds before centrifugation at 14,000xg, 16°C for 20 minutes. The supernatants were collected, and protein concentration was measured by BCA protein assay. The protein concentration was then adjusted to 1 µg/µL with 1xPBS+0.1%Tween20. Ten microliters of slurry protein A beads were added to the protein solution for 1 hour at 37°C to remove non-specific binding. The mixtures were transferred to a centrifugal filter (Thermo Fisher) and the flow-through (FT) was collected. Anti-GFP antibody (Chromotek) (25 µL) was mixed with the FT and incubated at 8°C for 16 hours before adding 25 µL of protein A beads. The mixtures were incubated at 25°C for 3 hours and then transferred to a centrifugal filter (Thermo Fisher). The flow-through (FT) was discarded at this time. The beads were washed with 400 µL of 1x PBS + 0.1% Tween 20 for three times, and then washed with 400 µL of 1x PBS for four times. The beads were collected and subjected to on-bead digestion. Briefly, beads were resuspended in 0.15% RapiGest SF Surfactant (Milford, MA, USA), 10 mM NaCl, and 10 mM ammonium bicarbonate. The mixture was subjected to probe tip sonication at a frequency of 20 kHz and 80% amplitude for 2 seconds. Subsequently, 2 mM of TCEP was added to the samples and incubated at 90°C for 15 minutes. After that, the samples were cooled down, and 10 mM IAA solution was added. The samples were incubated in the dark at room temperature for 45 minutes. After that, 50 ng/µl of trypsin in ammonium bicarbonate at a 1:50 w/w ratio was added, and the samples were incubated at 37°C for 4 hours. Lastly, 1% formic acid at a 1:10 v/v ratio was added to terminate the reaction, and the tryptic peptides were lyophilized before LC-MS/MS analysis.

### LC-MS/MS setting and protein searching for protein identification

The LC-MS/MS spectrum data were collected in the positive mode with an Orbitrap HF mass spectrometer combined with nano-LC system equipped with an EasySpray C18 column (Thermo Scientific ES903; 75 μm x 50 cm, 2.0 µm) using a previous protocol with minor modifications [52]. Briefly, mobile phase A consisted of 0.1% formic acid in water and mobile phase B consisted of 100% acetonitrile with 0.1% formic acid. Separation was conducted with a linear gradient of 5%–45% mobile phase B at a constant flow rate of 300 nL/min over a period of 115  minutes. The tryptic peptides were analyzed by applying a data-dependent acquisition method, followed by higher-energy collisional dissociation. Full scan mass spectra were acquired at an *m/z* ratio of 400 to 1600 with an AGC target set at 3×10^6^ ions, a resolution of 120k, and an injection time was 60 ms. MS/MS scanning was initiated when the automatic gain control target reached 3e^6^ ions and, a resolution of 60k, and injection time was 100 ms. Isolation windows were 1.6 *m/z*. Xcalibur software (Thermo Fisher) was utilized to automatically collect the mass spectra. Raw LC–MS/MS files underwent analysis using the Proteome Discoverer with the SEQUEST HT algorithm (Thermo Fisher), referencing the in-house protein database, adhering to specific criteria: strict trypsin specificity, up to two missed cleavages, a fixed carbamidomethyl modification on cysteine (+57.0215 Da) and a variable modification on methionine (+15.9949). The relative protein abundance was standardized using the software’s normalization algorithm. Proteins that had a FDR value < 1% were selected for further analysis.

### Single cell infection assay and fluorescence microscopy

To observe Churi infection morphology under fluorescence microscopy, 1/4 LB containing 1.2% agarose pads on concavity slides were prepared. Each pad contained 1 µg/mL FM4-64 for cell membrane staining, and 1 µg/mL DAPI for DNA staining [18,53]. Colonies of *P. aeruginosa* K2733 were resuspended in 1/4 LB broth before 5 µL of the suspension was inoculated onto the agarose pad. The K2733 strain is derived from PAO1 that multiple efflux pumps are knockout (ΔmexB, ΔmexX, ΔmexCD-oprJ, ΔmexEF-oprN) and is used in the microscopy experiment [29]. This improves cell staining with fluorescent dyes while identical results were obtained in both PAO1 and K2733 [29]. The slides were then incubated in a humid chamber at 30°C for 3 hours. After that, 5 µL of high titer Churi lysate was added onto the pads. A cover slip was placed on the pad before fluorescence microscopy. For localization profiling, recombinant *Pseudomonas* cells that contain fluorescence-tagged version of Churi proteins were grown on the agarose pads, supplemented with the suitable concentration of arabinose to induce phage gene expression for 3 hours at 30°C in the humid chamber prior to Churi infection. To observe the effect of gp335-Churi on bacterial cell morphology, overnight cultures of *P. aeruginosa* K2733 carrying gp335-, sfGFP-, and empty-pHERD30T were inoculated into LB broth, supplemented with gentamicin and different concentrations of arabinose. The cultures were rotated on a roller at 37°C for 3 hours. After incubation, the cells were centrifuged at 8,000 rpm for 1 minute. The pellets were resuspended and mixed with FM4-64 and DAPI mixture before added onto the agarose pads. The DeltaVision Spectris Deconvolution Microscope (Applied Precision, Issaquah, WA, USA) was used to visualize the cells. For static images, the cells were imaged for at least five stacks from the middle focal plane with 0.15 mm increments in the z-axis and, for time-lapse imaging, the cells were imaged from a single stack at the focal plane with the ultimate focusing mode. Microscopic images were further processed by the deconvolution algorithm in the DeltaVision SoftWoRx Image Analysis Program [15].

### Generation of gp335 mutant Churi with Cas13a

Plasmids were synthesized and cloned by Genscript (USA). The vector for all *P. aeruginosa* plasmids was pHERD30T (S6 Table). The plasmids were transformed at 2.5 kV electroporation into K2733 competent cells. *P. aeruginosa* K2733 that contains Cas13a expression vector pHERD30T-LbuCas13a with a guide targeting gp335-Churi were grown to OD_600_~0.5–0.8 in LB containing 15 μg/mL gentamicin and 0.2% arabinose. One-hundred microliters of the culture were infected with high titer Churi lysate and mixed with 5 mL of LB 0.35% agar containing 0.2% arabinose at 55°C. Lawns of *P. aeruginosa* expressing gp335-targeting Cas13a were grown on LB plates containing 15 μg/mL gentamicin. Single plaques of Churi mutants escaping from Cas13a activity were selected and streaked to purify at least three times using bacteria expressing gp335-targeting Cas13a as the host [30]. Finally, the targeted region on the Churi genome was amplified by PCR and the amplicon sequenced by Sanger sequencing to identify mutations and confirmed by whole genome sequencing. The phage lysate samples were sequenced by the SeqCenter (Pittsburgh, USA). After genomic extraction, whole genome sequencing was performed on an Illumina NovaSeq 6000 sequencer in one or more multiplexed shared-flow-cell runs, producing 2 x 151bp paired-end reads with coverage at 2,618. Demultiplexing, quality control, and adapter trimming were performed with bcl-convert1 (v4.1.5). The variant calling was performed using reference genome sequence information of Churi (Accession No. OM718002.1).

### Single-Step Time-to-Lysis

Single-step time-to-lysis was applied to measure the effect of gene mutation on mutant Churi lysis time point in real-time [31]. Briefly, *P. aeruginosa* strain PAO1-K2733 was grown in LB broth at 37°C to early log phase (OD_600_ ~0.25–0.3). High titer wild-type or mutant Churi was mixed with the culture at an MOI of 5 and incubated at 30°C for 20 minutes before adding 5 µM SYTOX Green Nucleic Acid Stain (Thermo Fisher). Next, 200 µL of each phage-bacteria mixture was transferred into a black-walled, clear-bottom 96-well plate (Costar). Fluorescence and optical density measurements were performed in a microplate reader (Tecan Infinite M Plex) at 30°C. Fluorescence measurements were taken with the following settings: λ-excitation = 504 nm; λ-emission = 537 nm; gain = 25; flashes per well = 5. Optical density measurements were taken with the following settings: λ-excitation = 600 nm; flashes per well = 5. Measurements were taken every 2 minutes for 45 cycles, to a maximum of 120 minutes post-infection. The plate was shaken for 20 seconds and waited for 20 seconds before measurements. Data analysis was done with Microsoft Excel. For each replicate, background fluorescence was subtracted, and values were divided by the maximum fluorescence signal as a fraction of maximal fluorescence equal to 1. Negative values due to photobleaching of the background prior to lysis were set to zero. The mean +/- the standard deviation (SD) across replicates was plotted and average time-to-lysis determined as the time point at which the mean fraction of maximal fluorescence reached 0.5 [30]. For complementation experiment, *P. aeruginosa* K2733 strain containing gp335-pHERD30T was incubated with 0.2% or without arabinose at 37°C before infected with gp335G2-2 mutant Churi.

## Statistical analysis

Statistical analysis was applied to investigate whether the mutation on gp335 affects Churi propagation. Briefly, early log-phase of *P. aeruginosa* K2733 was incubated with wild-type and gp335-mutant Churi lysates at an MOI of 5 for 60, 80, and 90 mpi. The mixtures were filtered, then diluted before spotted on the lawn of *P. aeruginosa* K2733. The experiments were performed in triplicates. The plaques were counted after incubation and the titers between wild-type and gp335-mutant Churi were statistically compared with paired samples *t*-test function (IBM SPSS statistics 28.0.0.0.). If P-values between the phage titers of wild-type and the mutant are less than 0.05, the mutation on gp335 is considered to statistically affect progeny propagation of Churi.

## Supporting information

S1 Fig Characteristics of phage Churi. (A) Raw images of Churi infection (10, 45, 60, 90, and 120 mpi). FM4-64 (red) represents bacterial cell membrane and DAPI (gray) represent DNA. Scale bar represents 2 µm. (B) sfGFP-gp285 (ChmA) morphology at 90 mpi of Churi infection against *P. aeruginosa*. Scale bar is 1 µm. (C) Bouquet counts of phage Churi during infection (60, 90, and 120 mpi) against *P. aeruginosa* K2733. n≥150 cells in each mpi.
(TIF)

S2 Fig Growth inhibitory activity test against *P. aeruginosa.* (A) Growth inhibition assay of gp335-Churi with different arabinose concentrations from 0.2 to 0.6% compared to empty-pHERD30T. (B) Optical density (OD_600_) of bacteria expressing empty-pHERD30T (negative control), and gp10-JJ01 (positive control) when the cells were induced with different concentrations of arabinose (0.2, 0.4, and 0.8%). Shaded error bar represents standard deviation (±SD) of n=6.
(TIF)

S3 Fig Raw images showing gp335 -sfGFP localization when *P. aeruginosa* is infected with Churi (15 to 120 mpi). Scale bar represents 2 µm.
(TIF)

S4 Fig Multiple alignment of amino acid sequences (aligned with Clustal Omega analysis) between gp335-Churi against its homologs (gp014-phiKZ and gp122-phiPA3). Black frames represent the conserved amino acid residues that gp014-phiKZ uses to interact with host ribosomes as previously reported [17].
(TIF)

S1 Table VIRIDIC analysis of similarity distance (%) and cluster tables between Churi and other nucleus-forming phages (OMKO1, phiKZ, phiPA3, and 201phi2-1) (PDF)

S2 Table Mass Spectrometry results of 31 non-virion phage proteins produced by Churi that were detected during early infection against *P. aeruginosa* (infection on plate). Abundance indicates the number of peptides counted. The grey-shaded rows indicate 19 proteins consistently detected in both infection models.
(PDF)

S3 Table Mass Spectrometry results of 129 non-virion phage proteins produced by Churi that were detected during early infection against *P. aeruginosa* (infection in broth). Abundance indicates the number of peptides counted. The grey-shaded rows indicate 19 proteins consistently detected in both infection models.
(PDF)

S4 Table List of all proteins of *P. aeruginosa* PAO1 proteins according to Sequest HT score ranking that were detected from Co-immunoprecipitation experiment with gp335-sfGFP using Mass spectrometry. (PDF)

S5 Table Amino acid sequence similarity (%) of gp335-Churi against all its homologs found in NCBI database (aligned with Clustal Omega analysis) (PDF)

S6 Table List of plasmid constructs that were used in this study. (PDF)

S1 Data Sheet 1: Raw data of One-step growth result of Churi against *P. aeruginosa* PAO1 for Fig 1C. Sheet 2: Raw data of Growth inhibition assay (real-time measurement by OD600) for Fig 2C–E. Sheet 3: Raw data of distribution plots corresponding to the kymograph show the different pattern of GFP intensity between (Fig 3E) sfGFP control and (Fig 3F) gp335-sfGFP. Sheet 4: Raw data of titer count (PFU/mL) in different minutes post-infection between wild-type (gp335WT) and gp335G2-2 mutant Churi as determined by spot test on *P. aeruginosa* K2733 lawns for Fig 5C. Sheet 5: Raw data of single-step time-to-lysis representing real-time bacterial cell lysis caused by the phage infection for Fig 5D–F.
(XLSX)
