# Supplementary figures and images for: A ribosome-interacting jumbophage protein associates with the phage nucleus to facilitate efficient propagation
Source: PLoS Pathog. 2025 Feb 24;21(2):e1012936. doi: 10.1371/journal.ppat.1012936 (PMC11849849; doi:10.1371/journal.ppat.1012936)

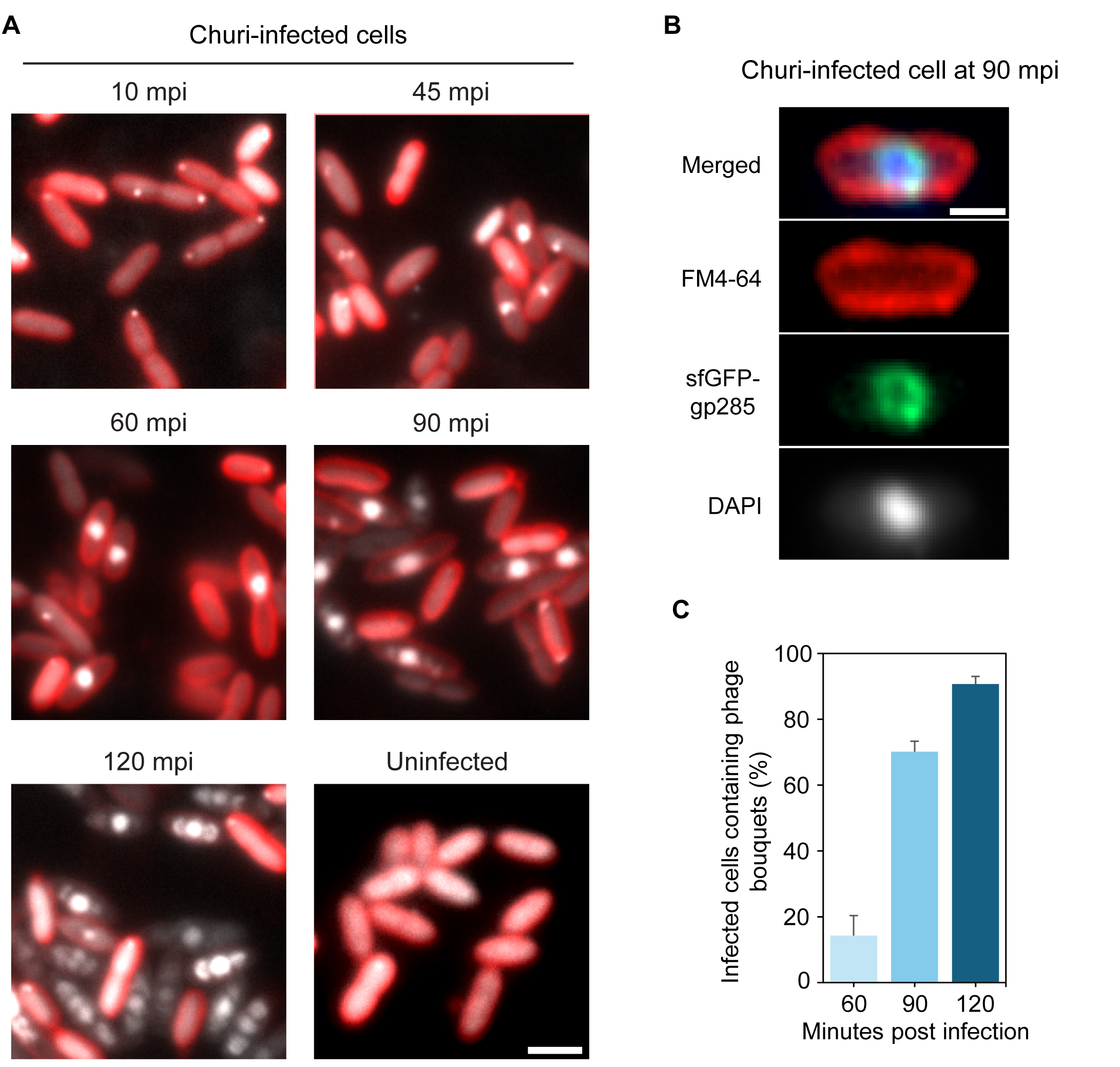

Supplement: S1 Fig — (A) Raw images of Churi infection (10, 45, 60, 90, and 120 mpi). FM4-64 (red) represents bacterial cell membrane and DAPI (gray) represent DNA. Scale bar represents 2 µm. (B) sfGFP-gp285 (ChmA) morphology at 90 mpi of Churi infection against P. aeruginosa. Scale bar is 1 µm. (C) Bouquet counts of phage Churi during infection (60, 90, and 120 mpi) against P. aeruginosa K2733. n≥150 cells in each mpi. (TIF) [file ppat.1012936.s001.tif]

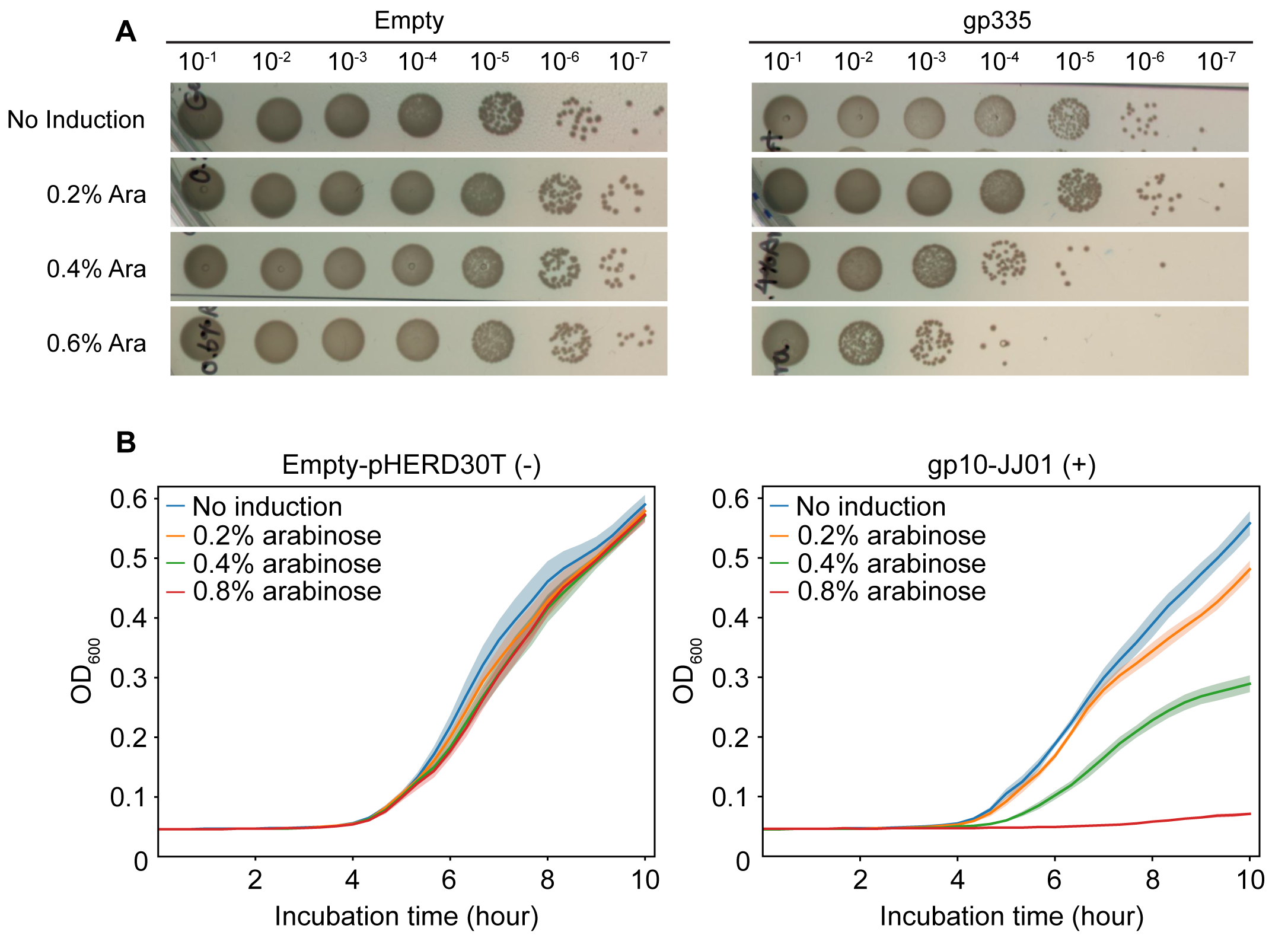

Supplement: S2 Fig — (A) Growth inhibition assay of gp335-Churi with different arabinose concentrations from 0.2 to 0.6% compared to empty-pHERD30T. (B) Optical density (OD600) of bacteria expressing empty-pHERD30T (negative control), and gp10-JJ01 (positive control) when the cells were induced with different concentrations of arabinose (0.2, 0.4, and 0.8%). Shaded error bar represents standard deviation (±SD) of n=6. (TIF) [file ppat.1012936.s002.tif]

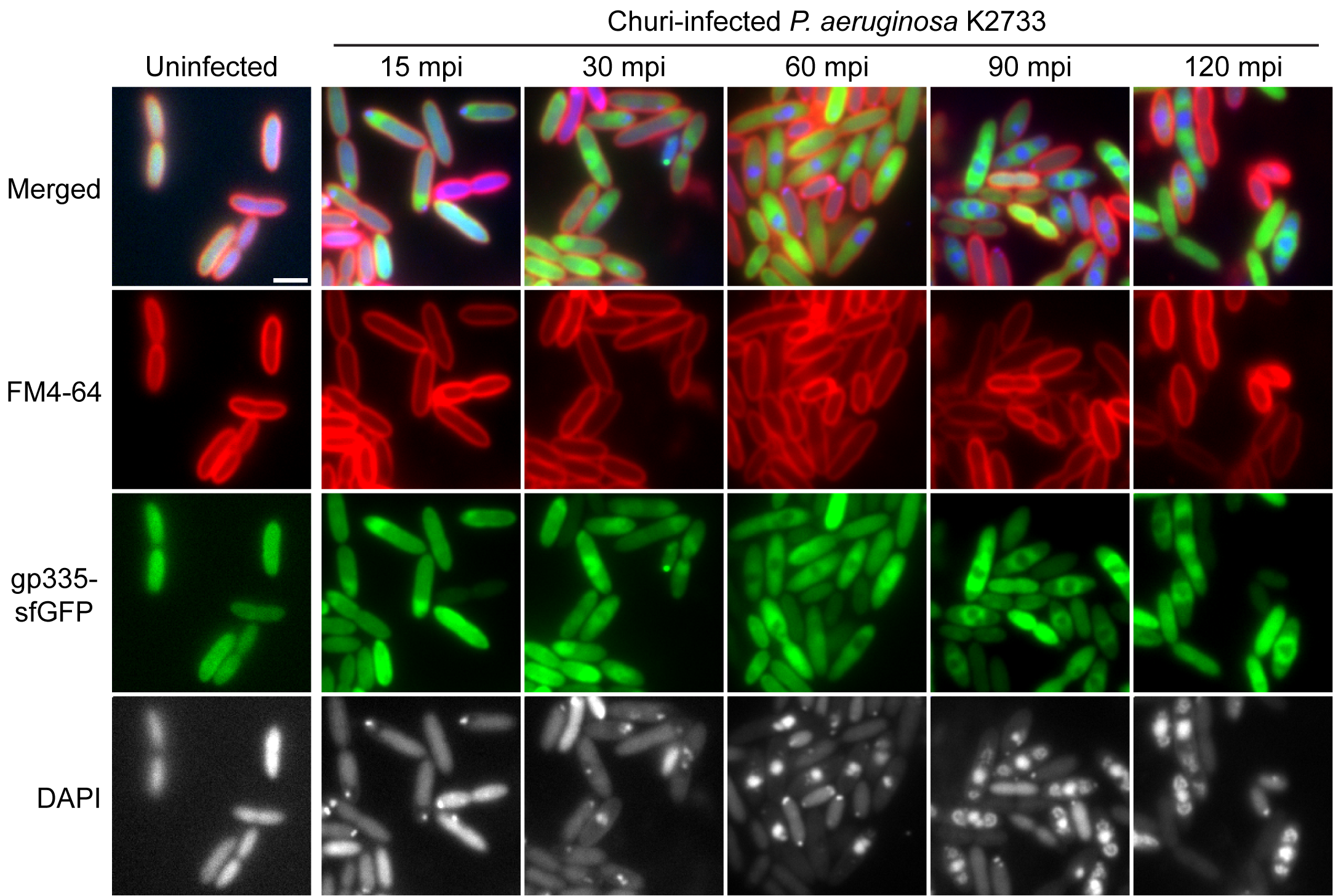

Supplement: S3 Fig — Scale bar represents 2 µm. (TIF) [file ppat.1012936.s003.tif]

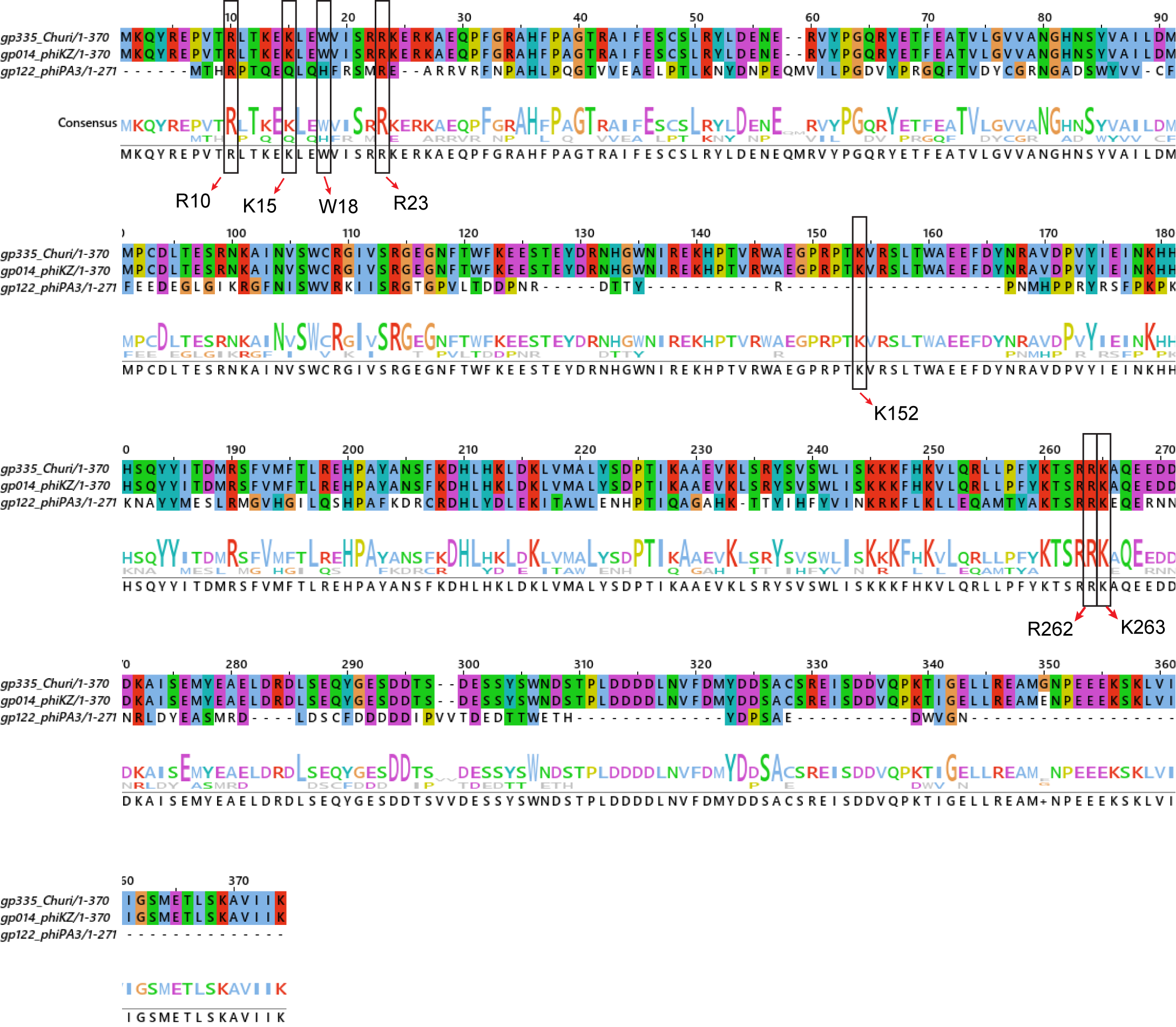

Supplement: S4 Fig — Black frames represent the conserved amino acid residues that gp014-phiKZ uses to interact with host ribosomes as previously reported [17]. (TIF) [file ppat.1012936.s004.tif]
