# Supplementary material for: A ribosome-interacting jumbophage protein associates with the phage nucleus to facilitate efficient propagation
Source: PLoS Pathog. 2025 Feb 24;21(2):e1012936. doi: 10.1371/journal.ppat.1012936 (PMC11849849; doi:10.1371/journal.ppat.1012936)
Supplement: S1 Table — (PDF) [file ppat.1012936.s005.pdf]

S1 Table. VIRIDIC analysis of similarity distance (%) and cluster tables between Churi and other nucleus-forming phages (OMKO1, phiKZ, phiPA3, and 201phi2-1)

**Genome**	Churi	OMKO1	phiKZ	201phi2-1	PhiPA3
Churi	100	94.2	94.2	12.8	18.3
OMKO1	94.2	100	94.4	12.5	18.2
phiKZ	94.2	94.4	100	12.3	18.5
201phi2-1	12.8	12.5	12.3	100	20.0
PhiPA3	18.3	18.2	18.5	20.0	100

**Genome**	**Species cluster**	**Genus cluster**
Churi	2	2
OMKO1	3	2
phiKZ	4	2
201phi2-1	1	1
PhiPA3	5	3
