# Supplementary material for: A ribosome-interacting jumbophage protein associates with the phage nucleus to facilitate efficient propagation
Source: PLoS Pathog. 2025 Feb 24;21(2):e1012936. doi: 10.1371/journal.ppat.1012936 (PMC11849849; doi:10.1371/journal.ppat.1012936)
Supplement: S2 Table — Abundance indicates the number of peptides counted. The grey-shaded rows indicate 19 proteins consistently detected in both infection models. (PDF) [file ppat.1012936.s006.pdf]

S2 Table. Mass Spectrometry results of 31 non-virion phage proteins produced by Churi that were detected during early infection against *P. aeruginosa* (**infection on plate**). Abundance indicates the number of peptides counted. The grey-shaded rows indicate 19 proteins consistently detected in both infection models.

Proteins	Function	Sum PEP Score	Coverage (%)	# Peptides	# PSMs	# Unique Peptides	Abundances (15 mpi)
**gp005**	Hypothetical protein	2.945	40	2	3	2	162.4
**gp039**	Hypothetical protein	1.171	6	1	15	1	54.2
**gp055**	Hypothetical protein	3.035	27	1	5	1	174
**gp059**	Hypothetical protein	2.523	6	1	26	1	98.6
**gp071**	Hypothetical protein	1.294	7	1	9	1	136
**gp094**	Hypothetical protein	1.425	2	1	22	1	48.4
**gp110**	Hypothetical protein	1.171	3	1	23	1	125.4
**gp119**	Hypothetical protein	1.537	4	1	37	1	82.5
**gp123**	Hypothetical protein	1.162	10	1	5	1	127.3
**gp126**	Hypothetical protein	2.026	10	1	1	1	51
**gp130**	Hypothetical protein	4.557	8	1	14	1	64.7
**gp135**	Hypothetical protein	6.008	19	1	5	1	139.7
**gp136**	Hypothetical protein	1.348	1	1	37	1	88.3
**gp147**	Hypothetical protein	1.681	3	1	36	1	73.9
**gp150**	Hypothetical protein	2.705	13	2	52	2	105
**gp177**	Hypothetical protein	5.032	4	2	17	2	105.1
**gp199**	Hypothetical protein	7.569	4	2	4	2	66.7
**gp202**	Hypothetical protein	1.168	5	1	9	1	178.8
**gp218**	Hypothetical protein	1.073	7	1	13	1	98.5
**gp234**	Hypothetical protein	1.52	1	1	4	1	86
**gp256**	Hypothetical protein	4.921	8	2	2	2	21.3
**gp269**	Hypothetical protein	13.486	6	2	7	2	35.3
**gp270**	Hypothetical protein	1.85	3	1	1	1	135.3
**gp271**	Hypothetical protein	1.335	4	1	58	1	31.6
**gp279**	Hypothetical protein	2.388	33	2	3	2	215.4
**gp287**	Hypothetical protein	2.811	2	1	16	1	95.4
**gp291**	Hypothetical protein	1.09	16	1	1	1	36.6
**gp325**	Hypothetical protein	2.784	20	1	3	1	72.9
**gp335**	Hypothetical protein	2.36	2	1	18	1	89.4

**gp352**	Putative ribonucleoside-diphosphate reductase	1.338	4	1	1	1	17.5
**gp354**	Hypothetical protein	1.961	28	1	3	1	158.2
