# Supplementary material for: A ribosome-interacting jumbophage protein associates with the phage nucleus to facilitate efficient propagation
Source: PLoS Pathog. 2025 Feb 24;21(2):e1012936. doi: 10.1371/journal.ppat.1012936 (PMC11849849; doi:10.1371/journal.ppat.1012936)
Supplement: S3 Table — Abundance indicates the number of peptides counted. The grey-shaded rows indicate 19 proteins consistently detected in both infection models. (PDF) [file ppat.1012936.s007.pdf]

S3 Table. Mass Spectrometry results of 129 non-virion phage proteins produced by Churi that were detected during early infection against *P. aeruginosa* (**infection in broth**). Abundance indicates the number of peptides counted. The grey-shaded rows indicate 19 proteins consistently detected in both infection models.

Proteins	Function	Sum PEP Score	Coverage (%)	# Peptides	# PSMs	# Unique Peptides	Abundances (15 mpi)
**gp003**	Hypothetical protein	9.705	23	3	25	3	32.2
**gp005**	Hypothetical protein	2.224	16	1	1	1	22.1
**gp007**	Hypothetical protein	12.205	29	4	38	4	23.9
**gp008**	Hypothetical protein	13.729	22	5	26	5	4.4
**gp012**	Hypothetical protein	10.417	31	3	30	3	73.9
**gp014**	Hypothetical protein	31.108	56	10	91	10	25.9
**gp017**	Hypothetical protein	6.982	14	4	11	4	51.7
**gp018**	Hypothetical protein	19.144	32	7	57	7	23.5
**gp019**	Hypothetical protein	6.213	21	3	18	3	31.5
**gp020**	Hypothetical protein	10.644	25	3	13	3	109.8
**gp021**	Hypothetical protein	11.141	22	3	11	3	15.9
**gp024**	Hypothetical protein	8.956	18	2	10	2	54.7
**gp028**	Hypothetical protein	19.427	40	7	24	7	40.8
**gp031**	Hypothetical protein	7.702	13	2	12	2	202.4
**gp035**	Hypothetical protein	12.079	31	1	21	1	176.3
**gp037**	Hypothetical protein	2.487	22	1	7	1	217.5
**gp042**	Hypothetical protein	9.283	22	3	31	3	44.2
**gp043**	Hypothetical protein	14.364	84	6	43	6	18.4
**gp044**	Hypothetical protein	2.073	10	1	5	1	129
**gp045**	Hypothetical protein	7.458	11	1	10	1	71.6
**gp046**	Hypothetical protein	8.581	21	2	13	2	23.5
**gp049**	Hypothetical protein	8.72	22	2	14	2	16.5
**gp052**	Hypothetical protein	8.954	38	4	15	4	229.8
**gp059**	Hypothetical protein	9.215	25	3	38	3	176.9
**gp062**	(p)ppGpp synthetase, RelA/SpoT family	10.344	23	4	11	4	43.7
**gp065**	Hypothetical protein	16.549	23	7	43	7	112.3
**gp066**	Hypothetical protein	7.222	25	3	18	3	20.6
**gp068**	Hypothetical protein	20.015	48	5	40	5	80.1
**gp072**	Hypothetical protein	7.819	21	3	26	3	37.2

**gp073**	Predicted nucleotidyltransferase	7.583	19	5	8	5	30.5
**gp074**	Putative thymidylate synthase	19.397	15	6	30	6	40
**gp076**	Hypothetical protein	6.245	30	3	14	3	151.6
**gp077**	Hypothetical protein	9.836	22	3	30	3	36.8
**gp078**	Hypothetical protein	2.275	9	1	6	1	77.4
**gp079**	Stringent starvation protein B	47.531	45	8	72	8	24.5
**gp088**	Hypothetical protein	13.233	56	3	25	3	243.4
**gp094**	Hypothetical protein	16.488	19	6	33	6	181.7
**gp098**	Hypothetical protein	17.81	45	6	44	6	41.6
**gp100**	Deoxycytidine triphosphate deaminase	7.738	21	3	19	3	159
**gp102**	Hypothetical protein	6.984	32	3	9	3	28.4
**gp108**	AAA family ATPase	46.333	39	11	79	11	20.6
**gp110**	Hypothetical protein	6.732	12	3	13	3	45.9
**gp111**	Hypothetical protein	10.325	32	4	24	4	42
**gp114**	Superfamily II DNA or RNA helicase	7.593	5	3	12	3	66.5
**gp122**	Hypothetical protein	14.395	56	6	27	6	11.6
**gp123**	Hypothetical protein	7.209	25	3	15	3	35.3
**gp124**	Hypothetical protein	11.768	32	4	20	4	73.8
**gp128**	Hypothetical protein	17.952	43	3	68	3	60.9
**gp129**	Thymidylate kinase	33.999	30	9	97	9	108.5
**gp130**	Hypothetical protein	8.499	35	4	35	4	138.2
**gp131**	Hypothetical protein	55.618	54	6	174	6	39.2
**gp132**	Hypothetical protein	12.69	60	4	32	4	28.8
**gp133**	Hypothetical protein	10.201	24	2	32	2	48
**gp134**	Hypothetical protein	19.982	27	5	26	5	70.4
**gp135**	Hypothetical protein	12.674	40	3	48	3	5.5
**gp138**	Hypothetical protein	7.948	4	2	9	2	62.2
**gp140**	Hypothetical protein	7.308	55	1	3	1	184.8
**gp143**	Head protease	21.093	30	5	27	5	45.2
**gp145**	Hypothetical protein	16.175	24	6	36	6	46.9
**gp146**	Putative exonuclease	8.968	21	3	22	3	157.1
**gp148**	Hypothetical protein	11.105	18	3	19	3	61.2
**gp150**	Hypothetical protein	21.347	26	4	26	4	69.5
**gp167**	RNase_HI_prokaryote_li ke	10.226	10	4	16	4	28.1

**gp170**	Hypothetical protein	21.902	21	9	57	9	27.3
**gp172**	Hypothetical protein	8.874	27	1	5	1	124.6
**gp173**	Hypothetical protein	2.346	5	1	5	1	38.8
**gp174**	Hypothetical protein	14.466	26	3	24	3	54.7
**gp175**	Hypothetical protein	6.202	11	2	9	2	31
**gp176**	Collagen-like protein	134.971	42	22	266	22	186.1
**gp177**	Hypothetical protein	79.107	36	18	182	18	201.1
**gp184**	Hypothetical protein	19.341	22	6	44	6	131.4
**gp187**	Hypothetical protein	16.647	29	5	27	5	10
**gp188**	Hypothetical protein	16.045	17	5	23	5	101.3
**gp189**	Hypothetical protein	11.454	20	6	16	6	27.6
**gp197**	Hypothetical protein	2.426	12	1	2	1	72
**gp199**	Hypothetical protein	84.169	26	13	222	13	134.1
**gp200**	Hypothetical protein	9.983	13	6	14	6	137.2
**gp207**	Hypothetical protein	9.188	45	3	18	3	105
**gp223**	Phage protein	2.515	10	1	7	1	88.9
**gp225**	Hypothetical protein	7.931	18	2	17	2	15.1
**gp226**	Hypothetical protein	8.455	21	3	15	3	60.5
**gp235**	Ribonuclease	56.556	31	19	141	19	134.3
**gp246**	Hypothetical protein	17.643	21	7	55	7	106.5
**gp249**	Kelch-like protein	7.09	9	3	10	3	83.1
**gp251**	Hypothetical protein	17.954	14	7	24	7	37.4
**gp253**	Hypothetical protein	10.11	10	3	7	3	215.6
**gp254**	Hypothetical protein	14.186	26	5	17	5	248.4
**gp255**	Hypothetical protein	27.837	52	7	80	7	85.9
**gp256**	Hypothetical protein	36.777	24	12	56	12	40
**gp262**	Hypothetical protein	11.118	9	5	16	5	78.4
**gp263**	DNA-directed RNA polymerase subunit beta	44.905	28	15	72	15	29.9
**gp264**	Chain C PHIKZ071 DNA-directed RNA polymerase	6.823	13	2	12	2	238.6
**gp265**	Hypothetical protein	12.926	12	4	19	4	232.2
**gp268**	Med15 subunit of Mediator complex protein	52.595	43	13	159	13	42.4
**gp269**	Hypothetical protein	48.608	28	10	164	10	143.5
**gp270**	Hypothetical protein	43.43	25	9	115	9	79.1
**gp271**	Hypothetical protein	53.58	47	9	92	9	59.8
**gp275**	Hypothetical protein	30.135	60	8	73	8	74.3

**gp278**	Hypothetical protein	7.333	27	2	14	2	21.8
**gp279**	Hypothetical protein	8.792	21	2	5	2	69.9
**gp280**	Hypothetical protein	7.763	23	3	11	3	30.3
**gp286**	Hypothetical protein	7.974	25	2	10	2	50.6
**gp288**	Hypothetical protein	17.368	28	5	39	5	21.4
**gp290**	Hypothetical protein	21.125	15	10	49	10	1.5
**gp298**	Hypothetical protein	17.891	20	7	29	7	60.2
**gp305**	Cys-based protein tyrosine phosphatase	8.674	24	3	14	3	4.8
**gp307**	Hypothetical protein	18.714	48	7	36	7	70.6
**gp308**	gp37/Dip protein	12.236	21	4	41	4	34.3
**gp309**	Hypothetical protein	26.716	42	8	51	8	28.2
**gp311**	Hypothetical protein	13.076	21	4	25	4	136.6
**gp313**	Hypothetical protein	13.252	18	4	38	4	8
**gp316**	Hypothetical protein	22.072	17	5	29	5	4.4
**gp325**	Hypothetical protein	20.211	48	3	47	3	60.4
**gp330**	Phage protein	9.267	42	4	20	4	63.8
**gp334**	Hypothetical protein	8.991	16	4	13	4	60.1
**gp335**	Hypothetical protein	23.703	27	10	62	10	120.4
**gp338**	Hypothetical protein	13.726	44	4	30	4	33.9
**gp340**	Methyltransferase type 11	6.171	10	1	10	1	15.9
**gp342**	Hypothetical protein	11.944	63	5	42	5	83.7
**gp343**	Hypothetical protein	7.636	32	3	16	3	7
**gp344**	Hypothetical protein	12.931	49	2	13	2	72.9
**gp347**	Hypothetical protein	10.746	12	2	10	2	145.2
**gp352**	Putative ribonucleoside-diphosphate reductase	21.787	12	7	34	7	137.6
**gp353**	Putative ribonucleotide reductase beta subunit	21.369	23	8	42	8	60.2
**gp354**	Hypothetical protein	17.013	45	4	28	4	223.6
**gp355**	Hypothetical protein	52.184	32	14	124	14	200.8
**gp356**	Hypothetical protein	2.182	5	1	6	1	68.5
**gp360**	Hypothetical protein	21.833	49	8	62	8	123.2
**gp362**	Hypothetical protein	2.237	10	1	6	1	33.7
