# Supplementary material for: A ribosome-interacting jumbophage protein associates with the phage nucleus to facilitate efficient propagation
Source: PLoS Pathog. 2025 Feb 24;21(2):e1012936. doi: 10.1371/journal.ppat.1012936 (PMC11849849; doi:10.1371/journal.ppat.1012936)
Supplement: S4 Table — (PDF) [file ppat.1012936.s008.pdf]

S4 Table. List of all proteins of *P. aeruginosa* PAO1 proteins according to Sequest HT score ranking that were detected from Co-immunoprecipitation experiment with gp335-sfGFP using Mass spectrometry.

Accession	Description	Coverage (%)	No. of Peptides	No. of PSMs	No. of Unique Peptides	No. of AAs	Score Sequest HT
Q9HVK3	Transcriptional regulator RtcR	4	1	49	1	531	126.97
Q9HVT2	Alpha-2-macroglobulin homolog	1	1	43	1	1516	113.16
Q9HWK6	Lysyl endopeptidase	16	5	23	5	462	57.25
Q9I0R3	Probable chemotaxis transducer	4	1	23	1	535	56.5
Q9I2Y0	Probable transcriptional regulator	3	1	20	1	901	54.3
Q9HW00	Probable peptidoglycan glycosyltransferase FtsW	7	1	15	1	399	44.61
Q9HWE0	50S ribosomal protein L22	12	1	14	1	110	41.42
Q9HVY3	30S ribosomal protein S9	32	3	16	3	130	40.61
Q9I6N6	Probable transcriptional regulator	16	1	15	1	158	36.43
Q9HXZ3	tRNA(Ile)-lysidine synthase	6	1	13	1	442	32.52
Q9HTY4	Acetylornithine deacetylase	7	1	12	1	384	32.49
Q9I194	Acyl-homoserine lactone acylase PvdQ	2	1	14	1	762	29.23
Q9HTN8	50S ribosomal protein L28	13	1	11	1	78	25.03
Q9I4U5	Antitoxin Xre/MbcA/ParS-like toxin-binding domain-containing protein	22	1	9	1	95	23.45
Q9HWU9	Probable dehydrogenase	13	1	7	1	229	20.15
Q9HWD6	50S ribosomal protein L4	24	2	6	2	200	20.04
O52759	30S ribosomal protein S4	9	1	6	1	206	17.05
Q9HWF2	30S ribosomal protein S5	17	2	7	2	166	16.67
P05384	DNA-binding protein HU-beta	32	2	6	2	90	16.29
Q9HVY8	Stringent starvation protein B	19	1	5	1	135	14.2
Q9I2N7	Streptomycin 3''-phosphotransferase	9	1	5	1	275	12.83
Q9HW35	BifA	2	1	5	1	687	9.87
P23189	Glutathione reductase	6	1	4	1	451	9.84
Q9HVH0	Probable oxidoreductase	2	1	3	1	943	7.61
P30718	Chaperonin GroEL	5	1	3	1	547	7.57
Q9HVQ5	Phosphoethanolamine transferase CptA	3	1	3	1	600	7.12

Q9I2J7	Dienelactone hydrolase	4	1	3	1	546	7.08
Q9I2M9	Helicase ATP-binding domain-containing protein	3	1	3	1	758	6.39
Q9HWE2	50S ribosomal protein L16	10	1	2	1	137	5.68
Q9I3Y9	MoaD/ThiS family protein	20	1	2	1	126	5.6
Q9I1A1	Probable aldehyde dehydrogenase	3	1	2	1	771	3.87
Q9I0D8	Probable transcriptional regulator	7	1	1	1	339	2.87
Q9HXR7	Single-stranded-DNA-specific exonuclease RecJ	4	1	1	1	571	2.63
Q9HXJ8	GTPase Der	4	1	1	1	493	2.62
Q9I3A9	TonB-dependent receptor	4	1	1	1	702	2.61
Q9HZX7	Uncharacterized protein	13	1	1	1	162	2.58
Q9I2H1	Probable hydroxylase large subunit	3	1	1	1	734	2.44
Q9HWA1	TPR repeat-containing protein PA4299	11	1	1	1	245	2.4
Q9HWB2	Probable transcriptional regulator	8	1	1	1	267	2.37
Q9HZF6	Uncharacterized protein	6	1	1	1	326	2.37
Q9I2I1	Cobaltochelatase subunit CobN	2	1	1	1	1281	2.35
Q9I3X2	UvrD-like helicase C-terminal domain-containing protein	3	1	1	1	711	2.33
Q9I5N6	Filamentous haemagglutinin FhaB/tRNA nuclease CdiA-like TPS domain-containing protein	1	1	1	1	4180	2.31
Q9HV59	Polyribonucleotide nucleotidyltransferase	3	1	1	1	701	2.31
Q9I3M9	Cytochrome c-type biogenesis protein CycH	4	1	1	1	407	2.18
Q9HZ55	Ribosomal large subunit pseudouridine synthase B	6	1	1	1	386	2.08
Q9I2Q6	Probable RNA methyltransferase PA1839	6	1	1	1	346	2.03
P06200	Hemolytic phospholipase C	3	1	1	1	730	2.01
Q9HT34	Uncharacterized protein	13	1	1	1	186	1.94
