# Supplementary material for: A ribosome-interacting jumbophage protein associates with the phage nucleus to facilitate efficient propagation
Source: PLoS Pathog. 2025 Feb 24;21(2):e1012936. doi: 10.1371/journal.ppat.1012936 (PMC11849849; doi:10.1371/journal.ppat.1012936)
Supplement: S5 Table — (PDF) [file ppat.1012936.s009.pdf]

S5 Table. Amino acid sequence similarity (%) of gp335-Churi against all its homologs found in ncbi database (aligned with Clustal Omega analysis)

Protein no. (organism)	Accession no.	Amino acid sequence similarity (%)		
		gp335 (Churi)	gp014 (phiKZ)	gp122 (phiPA3)
gp335 (Churi)	UNI71859.1	100	99.73	28.41
gp014 (phiKZ)	NP_803580.1	99.73	100	28.41
gp122 (phiPA3)	YP_009217202.1	28.41	28.41	100
gp159 (Psa21)	YP_010347708.1	26.34	26.34	22.22
gp175 (Phabio)	YP_010348144.1	28.34	28.34	28
Hypothetical protein (*Pseudomonas aeruginosa*)	WP_213905353.1	42.86	42.86	29.91
FLI59_33855 (*Pseudomonas aeruginosa*)	TQH44198.1	50.33	50.33	30.6
Hypothetical protein (*Pseudomonas aeruginosa*)	HDU8983371.1	43.25	43.25	30.77
Hypothetical protein (*Pseudomonas aeruginosa*)	WP_280034350.1	43.25	43.25	30.77
FLI59_34140 (*Pseudomonas aeruginosa*)	TQH42362.1	32.28	32.28	26.5
PPEV_gp099 (EL)	YP_418132.1	42.46	42.46	30.77
Hypothetical protein (IR-QUMS-PaBa1-GHS-2021)	UZV40030.1	42.86	42.86	30.77
FLI59_34350 (*Pseudomonas aeruginosa*)	TQH39766.1	35.71	35.71	28.92
Hypothetical protein (PA1C)	QBX32281.1	61.11	61.11	24.32
RVBP21_3890 (BRkr)	BEG72761.1	80.27	80.54	31.6
gp016 (fnug)	QJB22659.1	82.29	82.02	31.6
gp126 (vB_PaeM_PS119XW)	YP_010660866.1	80.38	80.65	31.6
Hypothetical protein (PA1C)	QBX32282.1	76.8	77.35	33.86
gp022 (KEN49)	WWV93476.1	80	80.27	29.55
Hypothetical protein (PA7)	QXN68698.1	79.73	80	29.55
gp144 (OMKO1)	USL86602.1	91.35	91.62	28.41
gp355 (SL2)	YP_009619895.1	97.84	98.11	28.41
Hypothetical protein (pPA-N1803-4At.2)	WAX23553.1	98.92	99.19	28.41
Hypothetical protein (ANB1)	WNV49983.1	99.19	99.46	28.03
gp235 (PA02)	BBI55908.1	99.19	99.46	28.41
gp270 (NK1)	BDR26786.1	99.46	99.73	28.41
gp360 (Brmt)	BDR24870.1	99.46	99.73	28.41
LZK84_16440 (*Pseudomonas aeruginosa*)	MCT4994229.1	100	99.73	28.41
Hypothetical protein (*Pseudomonas aeruginosa*)	WP_015968824.1	99.73	100	28.41
