# Supplementary material for: A ribosome-interacting jumbophage protein associates with the phage nucleus to facilitate efficient propagation
Source: PLoS Pathog. 2025 Feb 24;21(2):e1012936. doi: 10.1371/journal.ppat.1012936 (PMC11849849; doi:10.1371/journal.ppat.1012936)
Supplement: S6 Table — (PDF) [file ppat.1012936.s010.pdf]

S6 Table. List of plasmid constructs that were used in this study.

Backbone vector	Insert	Strain No.
pHERD30T	sfGFP	WW1211
	JJ01-gp10	WW1258
	Churi-gp005	WW1080
	Churi-gp059	WW1119
	Churi-gp094	WW1178
	Churi-gp110	WW1179
	Churi-gp123	WW1181
	Churi-gp130	WW1121
	Churi-gp135	WW1085
	Churi-gp150	WW1109
	Churi-gp177	WW1183
	Churi-gp199	WW1184
	Churi-gp256	WW1186
	Churi-gp279	WW1112
	Churi-gp325	WW1149
	Churi-gp335	WW1235
	Churi-gp354	WW1087
	phiKZ-gp014	WW1256
	phiPA3-gp122	WW1257
	sfGFP-Churi-gp285 (ChmA)	WW1047
	Churi-gp335-sfGFP	WW1221
	Churi-gp335-guide-2 (CRISPR-Cas13a system)	WW1045 (Pogliano's lab)
	Churi-gp335G2-2-sfGFP (mutant Churi-gp335)	WW1052 (Pogliano's lab)
